# Oxidation States: Intrinsically Ambiguous?

**DOI:** 10.1021/acscentsci.4c00825

**Published:** 2024-06-25

**Authors:** Isaac F. Leach, Johannes E. M. N. Klein

**Affiliations:** † Molecular Inorganic Chemistry, Stratingh Institute for Chemistry, University of Groningen, Nijenborgh 3, 9747 AG Groningen, The Netherlands; ‡ Zernike Institute for Advanced Materials, University of Groningen, Nijenborgh 3, 9747 AG Groningen, The Netherlands

## Abstract

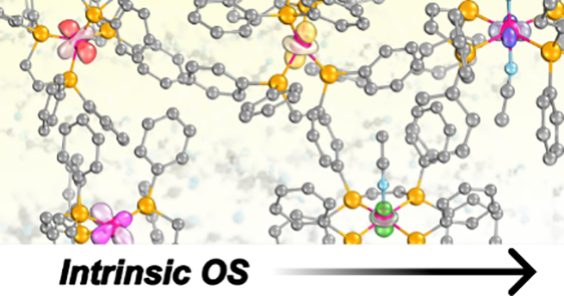

The oxidation state (***OS***) formalism is a much-appreciated good in chemistry, receiving wide application. However, like all formalisms, limitations are inescapable, some of which have been recently explored. Providing a broader context, we discuss the ***OS*** and its interpretation from a computational perspective for transition metal (TM) complexes. We define a broadly applicable and easy-to-use procedure to derive ***OS***s based on quantum chemical calculations, via the use of localized orbitals, dubbed the Intrinsic ***OS***. Applying this approach to a cobalt complex in five ***OS***s, isolated by Hunter and co-workers (Inorg. Chem.2021, 60, 1744534813328 10.1021/acs.inorgchem.1c03020), we find that the calculated Intrinsic ***OS*** matches the formal ***OS***, consistent with the experimental characterization. Through analysis of the delocalized orbitals, the ligand field of the Co(III) complex is found to be “inverted”, despite every cobalt–ligand bond being classically dative from the localized perspective—a bonding scenario very similar to that of [Cu(CF_3_)_4_]^−^. This is not atypical but rather a natural consequence of these metals bonding in the high-valent region, and we propose a more restrictive definition of (locally) inverted bonding. Additionally, two bonding descriptors within the Intrinsic Bonding Orbital (IBO) framework (σ-gain and π-loss) are introduced, which enable facile quantification of electron-sharing covalency across a broad range of TM complexes.

## Abstract 2

Intrinsic oxidation states are introduced, complimenting IUPAC’s ***OS*** definition and revisiting inverted bonding by broadly examining the role of ambiguity in ***OS*** assignment.

## Introduction

The oxidation state (***OS***, defined in Table 1), a long-standing and much-used molecular descriptor, has been enthusiastically revisited in recent decades.^1−40^ In this context, the [Cu(CF_3_)_4_]^−^ molecule has played an important role shaping the discourse;^1−14^ the ongoing debates about its electronic structure often revolve around the d^8^ Cu(III) classical Werner vs d^10^ Cu(I) inverted ligand field (ILF, see definition in Table 1) descriptions. In 2019, Lancaster and co-workers concluded that not only this system,^5^ but all formal Cu(III) centers,^7^ are best described as d^10^ Cu(I), calling for a “breakdown of [the] oxidation state formalism”.^7^ This sentiment is shared and debated in Hoffmann et al.’s 2016 review of ILFs,^6^ after which a flurry of works reported ILFs in other transition metal (TM) complexes.^a^ The sheer volume of such reports casts doubt on whether ILFs are unexpected or “atypical”. Indeed, several groups have shown, through computational and spectroscopic methods,^9,11,12^ that the electronic structure of [Cu(CF_3_)_4_]^−^ is consistent with a d^8^ Cu(III) center combined with significant electron-sharing covalent character in its metal–ligand σ-bonds. Since each of the Cu–CF_3_ σ-bonds is polarized toward the carbon center,^9−11^ it is difficult to justify the use of the term “inverted” from the localized point of view. However, we recently showed that the cumulative effect of these four bonds is the recovery of ca. two electrons worth of density by the metal.^10^ Our proposed *quasi-d*^*10*^ description of [Cu(CF_3_)_4_]^−^ serves as a bridge between these different views, one that does not require discarding the oxidation state formalism to cross, a suggestion that has been described as “interesting”^39^ by some, and as “unnecessarily cumbersome”^43^ by others–who noted that a *quasi-d*^*10*^ configuration is “a d^8^ configuration with a d-count approaching ten”.^43^ While we agree with this summary, it shows an evolution from earlier claims of “a 3d^10^ ground state electronic configuration [. . .] in [Cu(CF_3_)_4_]^1–^”.^5^ In our view, the introduction of such nuance results from the fact that the community is still moving toward a consensus on how to conceptualize these types of, sometimes ambiguous, bonding scenarios. Broadly speaking, this shift (from an “either/or” approach to one that is more comfortable with some intrinsic ambiguity) is encapsulated in a recent essay from Norman and Pringle, which highlights and addresses some of the “fundamental ambiguities associated with how a d^*n*^ number is determined”.^39^

**Table 1:** Definitions of Terms Relevant to (Intrinsic) ***OS***s

No.	Term	Definition	Reference
1	***OS*** of a metal (M) in a transition metal complex	***OS***_M_ = *N* – *n*, where *N* is the number of valence electrons and *n* is the number of d-electrons of a metal in a d^*n*^ configuration	IUPAC report from Karen et al.^18^
2	d^*n*^ of a metal in a transition metal complex	“The d^*n*^ number of a transition metal complex is assigned based on the number of electrons that occupy the frontier orbitals, which have the same symmetry as metal d-orbitals.”	Norman and Pringle^39^
3	Intrinsic ***OS***	The ***OS*** of an atom in a molecule or complex, as derived within the IBO framework. For a TM complex, the Intrinsic ***OS*** of the metal derives from the intrinsic d-configuration and the formula for ***OS*** in entry #1 of this table	This work
4	Intrinsic d-configuration	The number of electrons in occupied IBOs of an atom in a molecule which have the same symmetry as metal d-orbitals, i.e., local δ-symmetry	This work
5	Intrinsic Bonding Orbital (IBO)	A type of generalized Pipek–Mezey localized orbital	Knizia^49^
6	Intrinsic Atomic Orbital (IAO)	The flexible (polarizable) atom-centered functions that constitute the IBOs	Knizia^49^
7	Locally inverted σ-bond	A reversibly polarized bond, as expected from consideration of atomic electronegativity (as per IUPAC’s 2016 ***OS*** definition), and as judged by the charge distribution in a localized orbital, e.g., an IBO	This work
8	Inverted Ligand Field (ILF)	In a high-valent TM complex, an “inverted ligand field arises when [the σ-antibonding orbital] Ψ* has predominantly ligand orbital character”, i.e., when the “metal-localized molecular orbitals are located at lower energy relative to the [ligand-localized orbitals]”	Lancaster;^5^ see also Hoffmann^6^
9	*Quasi-d*^*n*^ configuration	A bonding scenario in which the central metal ion in a transition metal complex gains approximately two electrons worth of density via electron-sharing covalent bonding	Leach et al.;^10^ see also Trifonova et al.^73^

Since orbitals are typically optimized *variationally*, the canonical orbitals (used in Density Functional Theory, DFT, to calculate the energy via the Kohn–Sham operator) are a convenient choice. These orbitals (which include the familiar HOMO and LUMO) are readily connected to energetic transitions and thus find applications in analysis of, e.g., UV–vis and X-ray absorption spectra.^44−48^ On the other hand, complications can arise when they are used to analyze bonding as they are generally delocalized over the entire molecule (particularly in the valence space, near the Fermi level). All the electrons described by a (Kohn–Sham) wave function are indistinguishable; they are in a sense constantly interchanging with all the other electrons. This is at odds with most chemists’ conceptualization of electrons as spatially localized to certain regions of a molecule, e.g., within bonds in Lewis structures. This difference can be reconciled by the application of localized orbitals.^49−51^

The abundant examples of ILFs lead us to ask

- What do “typical” ***OS***s of TM complexes look like?
- How common are ILFs?
- When is a bonding scenario *normal*, when is it *inverted*?
- Is electron-sharing covalency not simply an intrinsic feature of high ***OS*** TM complexes?

To answer these questions, we turn toward a first row TM: cobalt. Naturally earth-abundant, cobalt today finds many applications in homogeneous catalytic transformations, e.g., hydrogenations,^52^ functionalization of alcohols and carbon dioxide,^53−55^ electrochemical oxidation of water,^56^ and selective oligomerization of ethene.^57−59^ TM complexes of cobalt not only play a key role in contemporary research but also did so back when many of our modern chemical ideas were still in their infancy. For example, it was through his seminal work (which included investigations into cobalt complexes)^60,61^ that Werner, sometimes dubbed the “*inorganic Kekulé*”, pioneered concepts in coordination chemistry.^62−64^

**Figure 1:**
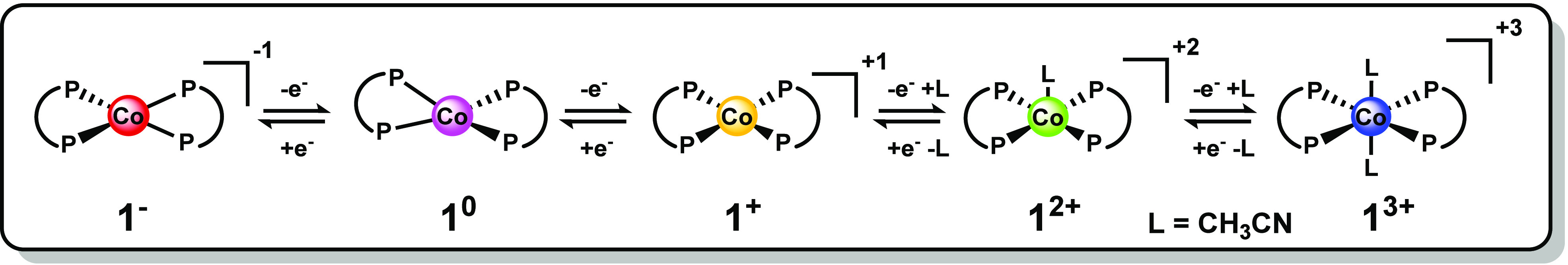
Complex **1**, with a cobalt–phosphine unit isolated in five oxidation states: **1**^***x***^, where *x* = [−1, 0, 1, 2, 3] conveniently is equal to both the total charge of the complex and the ***OS*** of cobalt.

In 2021, Hunter and co-workers isolated a cobalt complex, **1**, in five oxidation states featuring an almost uniform ligand framework (differing only by the addition of axially bound neutral solvent molecules, Figure 1).^65^ Furthermore, they demonstrated that the bidentate phosphine ligands, *cis*-1,2-bis(diphenylphosphino)ethylene, are both redox inactive. This important finding was substantiated by structural (X-ray diffraction) and spectroscopic data, which allowed assignment of all of the ***OS*** changes to the metal. This system therefore provides an ideal framework to examine the interplay between metal ***OS***s and metal–ligand bonding across the high- and low-valent extremes, uncomplicated by concerns of ligand redox activity.

## Results and Discussion

We optimized the geometries of **1** in all ***OS***s with the composite “Swiss army knife” DFT method r^2^SCAN-3c,^66^ as implemented in the ORCA code,^67,68^ starting from the reported crystallographic coordinates.^65^ For full computational details, see Supporting Information. The geometries of **1**^**x**^ obtained via single crystal X-ray diffraction by Hunter and co-workers,^65^ follow the predictions from crystal field theory (CFT), for the ideal geometries of low spin d^10^ to d^6^ complexes. Key structural parameters of the DFT optimized and previously reported experimental geometries show close agreement (Table S2).

**Figure 2:**
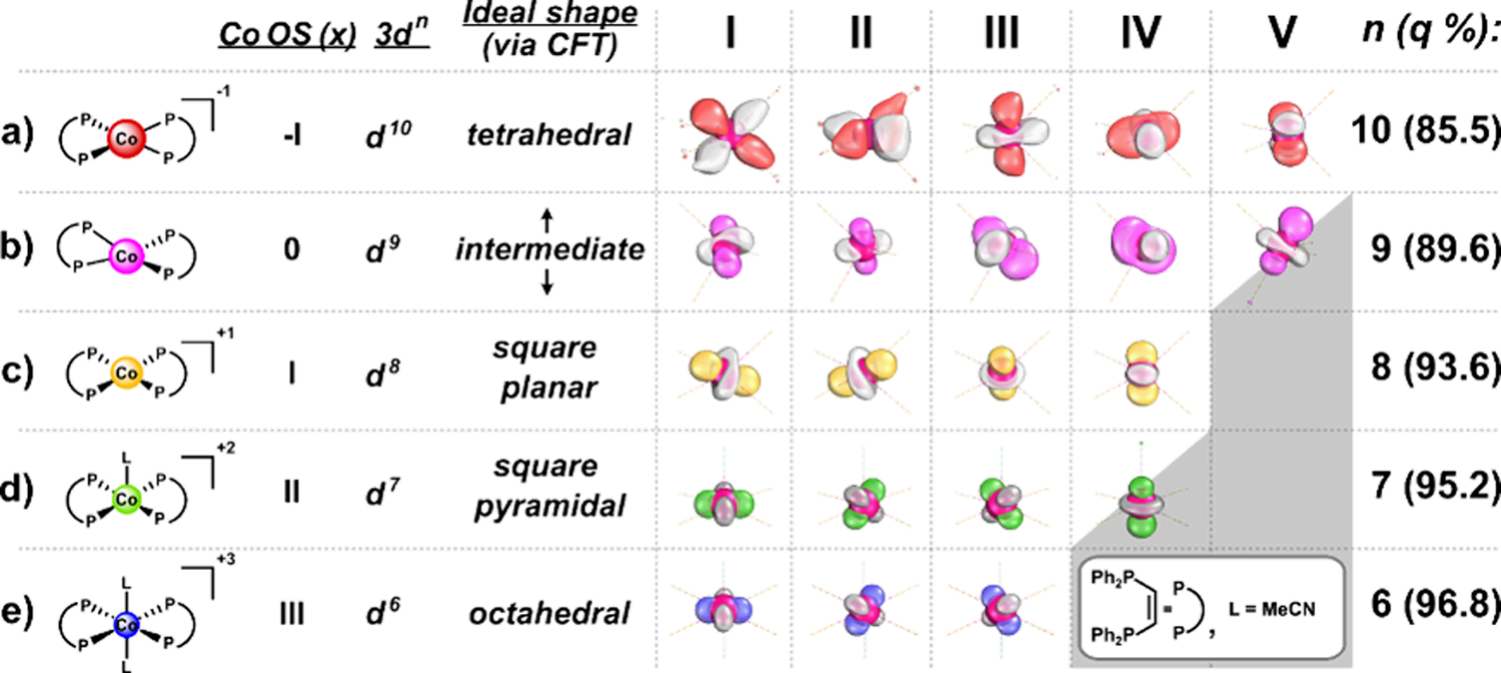
Cobalt–phosphine complex, **1**, with the corresponding formal d-configurations (3d^*n*^), ideal coordination geometry as predicted by crystal field theory (CFT), calculated intrinsic d-configurations (*n*), and individual d-orbitals represented by Intrinsic Bond Orbitals (IBOs, **I**–**V**) with their average IAO Co% (*q*). All orbitals are doubly occupied, apart from the half-gray boxes, in (b) **V** and (d) **IV**, which indicate singly occupied orbitals, and empty gray boxes, which indicate vacancies. Isosurfaces are rendered in IboView to enclose 70% of their electron density. Calculated with PBE0/def2-TZVP//r^2^SCAN-3c.

Although the (metal’s) calculated atomic partial charge is sometimes used as an electronic descriptor to assign ***OS***, this practice is rarely informative.^23−25^ Indeed, across the five experimentally established cobalt ***OS***s in **1**, the metal’s Intrinsic Atomic Orbital (IAO, defined in Table 1) partial charge (calculated with PBE0^69^/def2-TZVP^70^) varies by less than one electron (0.69 *e*, see Table S12). Here we note that within molecular orbital theory, one may choose (via rotations) from a set of reference frames to represent a 3*N*-dimensional wave function (where *N* is the number of electrons) as a set of *N* 3-dimensional one-electron functions, the *orbitals* (Figure S1). All such bases (or sets of orbitals) comprise the mathematically identical wave function and therefore share all measurable quantities (eq S1).^71^ One such basis, suitable for the analysis of intricate bonding scenarios, is provided by the localized Intrinsic Bond Orbitals (IBOs, see Table 1). Here the IBOs allow us to establish the *intrinsic d-configuration* (defined in Table 1) of these metal centers (*n*), by simply counting the valence IBOs with δ-symmetry that have a metal contribution of >70% as we have described elsewhere.^b^^,^^10,72−74^ Thus, a chemically intuitive picture quickly emerges (Figure 2, right). From this localized point of view, the derived intrinsic d-configuration agrees not only with the ***OS*** formalism^16−18^ but also with the experimental observations made by Hunter and co-workers.^65^ We also note that the calculated intrinsic d-configuration is compatible with Norman and Pringle’s recently refined definition of the d^*n*^ number (defined in Table 1).^39^

Examining Figure 2 (which is drawn to scale) more closely, we may notice that the d-orbitals in the high-valent regime of **1** (Figure 2, bottom) appear smaller than those in the low-valent regime (Figure 2, top). Some of the d-orbitals of **1**^**–**^ (Figure 2a, **I** and **II**) have lobes with pointy ends, of opposite phase, above and below the interatomic axes—already hinting at π-backbonding. The d-orbitals in **1**^**3+**^ (Figure 2e) are better localized onto cobalt than those of **1**^**–**^ (Figure 2a), and this observation can be formalized by a comparison of the average IAO Co% of these IBOs (*q*). Indeed, *q*(**1**^**3+**^) = 97% (1.94 *e*) (i.e., the metal center accounts for almost all of the electron density of these three orbitals), while *q*(**1**^**–**^) = 86% (*just* 1.71 *e*). The decrease in the average metal contribution to each d-orbital mirrors the increasing intrinsic d-configuration: *n*(**1**^**3+**^) = 6, *n*(**1**^**–**^) = 10. The more delocalized d-orbitals in **1**^**–**^ ultimately result from electron–electron repulsion, which is expected to be stronger at such a low ***OS***, where this region of Hilbert space with the same local symmetry as the metal’s d-orbitals is so crowded. This observation is best quantified by taking the difference between the metal’s total contribution to the d-orbital manifold, and the intrinsic d-configuration: π-loss = Σ*q*_π-IBO_(M) – *n*. For high-valent **1**^**3+**^, the π-loss is minimal (Σ*q*_π-IBO_(Co) – 6 = – 0.19 *e*), while it is maximized in low-valent **1**^**–**^ (Σ*q*_π-IBO_(Co) – 10 = – 1.45 *e*). The π-loss of the other ***OS***s in **1** varies smoothly between these extremes (Table S13).

**Figure 3:**
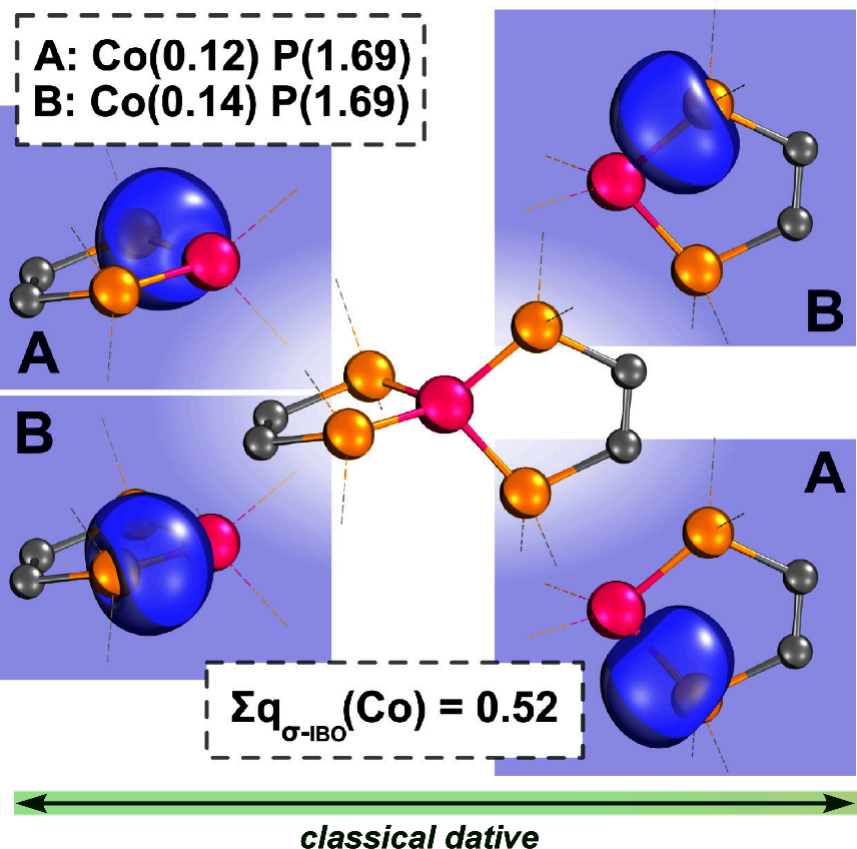
Doubly occupied metal–ligand bonding orbitals of **1**^**–**^, and their charge distributions, calculated with PBE0/def2-TZVP//r^2^SCAN-3c. Ligand side groups are omitted for clarity; isosurfaces are rendered in IboView to enclose 80% of their electron density. All orbitals shown are σ-IBOs with accompanying IAO charges.

**Figure 4:**
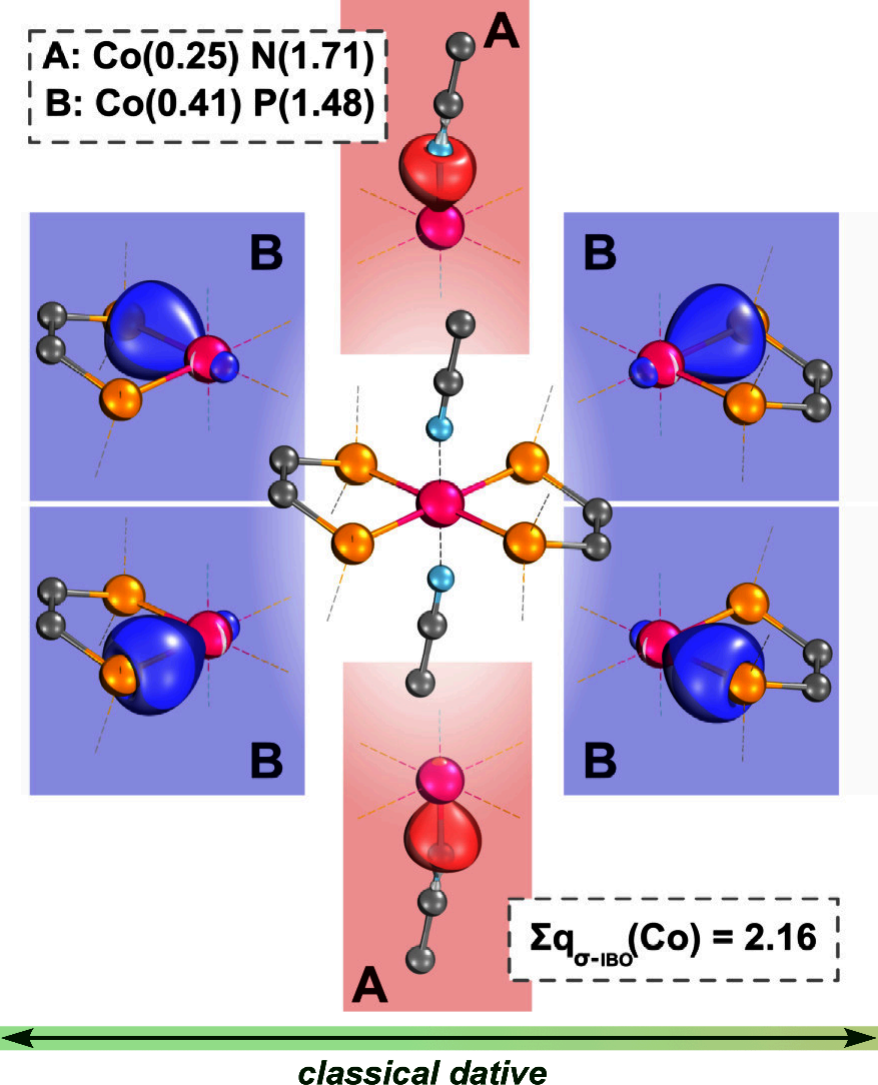
Doubly occupied metal–ligand bonding orbitals of **1**^**3+**^, and their charge distributions, calculated with PBE0/def2-TZVP//r^2^SCAN-3c. Ligand side groups are omitted for clarity; isosurfaces are rendered in IboView to enclose 80% of their electron density. All orbitals shown are σ-IBOs with accompanying IAO charges.

Turning our attention to the σ-bonding in the low-valent extreme (**1**^**–**^), we see that the metal center here forms four almost equivalent dative σ-bonds with >80% contributions from the phosphine moieties (A and B in Figure 3). In a similar manner as we have defined the π-loss (vide supra), and as we have previously shown in several coinage metal systems,^10,73−78^ we can quantify the cumulative amount of electron density gained by the metal through the σ-bonds by summing its contributions to these orbitals: σ-gain = Σ*q*_σ-IBO_(M). Here in the low-valent extreme, the σ-gain is minimal (Σ*q*_σ-IBO_(Co) = 0.52 *e*, Figure 3). This is, of course, contrasted by the high-valent case of **1**^**3+**^, where the σ-gain is maximal (Σ*q*_σ-IBO_(Co) = 2.16 *e*, Figure 4). This expected result is a numerical reflection of the chemically intuitive idea that as a metal’s ***OS*** increases, it forms more electron-sharing bonds with its ligands. Notably, the σ-gain of **1**^**3+**^ (2.16 *e*) is even greater than that of [Cu(CF_3_)_4_]^−^ (1.83 *e*),^c^^,^^10^ which has often served as a paradigmatic example of a complex with an ILF. Extending our perspective of [Cu(CF_3_)_4_]^−^ to **1**^**3+**^, we may describe this cobalt center as in a quasi-d^8^ configuration, since ∼2 *e* worth of density is recovered through σ-bonding. We note here that a quasi-d^*n*^ configuration certainly does not imply a physical d^*n*^ configuration but rather signifies (to the nearest integer) the increased level of electron-sharing metal–ligand bonding.

**Figure 5:**
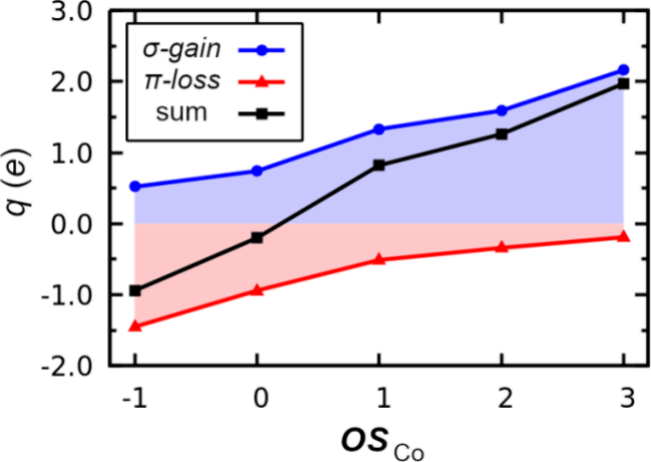
σ-gain (blue), π-loss (red), and their sum (black), versus the oxidation state of cobalt in **1** (***OS***_Co_) calculated with PBE0/def2-TZVP//r^2^SCAN-3c. For clarity, the regions between *q* = 0 and σ-gain/π-loss are shaded.

**Figure 6:**
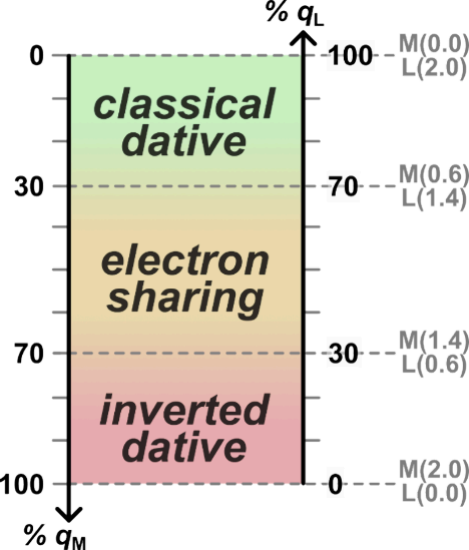
Spectrum of covalency, relating metal (M) and ligand (L) charge contributions (*q*) to localized orbitals with bonding regions, using a 70% ownership criterion.

The interplay between σ-gain and π-loss can be further examined by plotting these quantities versus ***OS*** (Co) for **1** (Figure 5). These data reveal that across the five ***OS***s of **1**, there is a smooth and continuous transition from the π-dominated bonding of **1**^**–**^ to the σ-dominated bonding of **1**^**3+**^, nicely reflecting discussions from Hoffmann et al. of “a continuous path from normal ligand field to inverted ligand field”.^6^ Despite the high cumulative amount of electron-sharing character in **1**^**3+**^ (σ-gain = 2.16 *e*), cobalt’s contribution to the individual metal–ligand bonds does not exceed 0.42 *e* (Figure 4). Indeed, every cobalt–ligand bond in **1** (in all five ***OS***s) is classically dative since the metal contributions are <0.6 *e* (Figure 6). Furthermore, the calculated intrinsic d-configuration matches the d-configuration predicted by the ***OS*** formalism in each case (Figure 2). It is therefore inappropriate to label the bonding of cobalt in **1** as “inverted”. However, we would have been led to that conclusion if we had applied Lancaster’s (delocalized) computational definition of an ILF (Table S13).^d^^,^^5,7^ Instead, in the localized framework provided by the IBOs, we can see that as the ***OS*** of **1** increases, the intrinsic d-configuration of the metal center changes as expected. The resultant large and unfavorable charge buildup at the metal center is of course mitigated through the σ- and π-bonding channels. This intuitive effect, which many chemists have already internalized, has been described in other contexts (from a more condensed matter physics perspective, in the study of semiconductors) as “charge self-regulation”.^23^ Indeed, these considerations often lead authors to refer to Pauling’s principle of electroneutrality, which dates back over seven decades and states that the electronic structure of a molecule will adjust itself to minimize the magnitude of atomic charges.^7,25,79,80^ The results presented here show explicitly how this principle manifests across low- and high-valent metal complexes without a breakdown in the ***OS*** formalism. In other words, there is no “rift between formalism and scrutable electronic structure”^7^ in either **1** or [Cu(CF_3_)_4_]^−^.^10^ Crucially, the intrinsic electronic structure of the formal Co(III) complex (**1**^**3+**^) is manifestly different from the Co(I) scenario (**1**^**+**^) (Figure 2c vs Figure 2e). Relabeling the former as *physically* Co(I) (because its σ-gain ≈ 2 *e*) would distort the natural trends in electronic structure that clearly emerge when this complex traverses the oxidation state terrain (Figures 2 and 5). Similarly for [Cu(CF_3_)_4_]^−^, we emphasize that the presence of an ILF is not itself sufficient to justify a *physical* Cu(I) assignment, echoing similar remarks from Geoghegan et al.^12^

The high total electron-sharing covalency (σ-gain ≈ 2.2 *e*) of **1**^**3+**^ and pronounced π-backbonding (π-loss ≈ 1.5 *e*) of **1**^**–**^ are natural consequences of these complexes lying near the limits of the high- and low-valent regimes on cobalt’s spectrum of accessible ***OS***s. More generally, we can classify the bonding type of a localized metal–ligand bond orbital by applying some threshold value to define a boundary between electron-sharing and dative covalent interactions. Necessarily, drawing a hard line on a gradual spectrum will involve a degree of arbitrariness, but we follow Neese and co-workers, who suggested a ∼ 70% cutoff value as a “useful operative criterion” for ownership of an electron pair in a localized orbital.^81^ As these authors noted, difficult ***OS*** assignments often arise near hard boundaries. As such, one should not tie oneself to the chosen cutoff, but rather carefully evaluate edge cases with thoughtful comparison. We also note that choosing an isosurface value such that 70–80% of a localized orbital’s density is contained within (as is done in IboView by default)^82^ is a useful way to rapidly judge bond characters via visual inspection.

**Figure 7:**
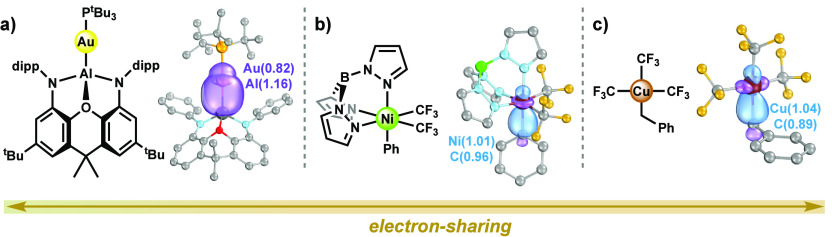
Examples of (organometallic) complexes reported by (a) Hicks et al.,^84^ (b) Bour et al.,^85^ and (c) Paeth et al.,^86^ with electron-sharing metal–ligand bonds. Shown alongside are the σ-IBOs of relevance, and their IAO charges, calculated with PBE0/def2-TZVP. “dipp” is 1,3-di(4-imidazolinophenoxyl)propane.

The classical dative σ-bonds in **1** are contrasted by several examples of organometallic complexes featuring highly electron-sharing covalent σ-bonds (with metal contributions ∼1 *e*) that have captured our attention recently (Figure 7):^10,74,83^ (a) the aluminyl–gold bond in (NON)Al–Au–P^t^Bu_3_ (where NON is the chelating tridentate ligand 4,5-bis(2,6-diisopropylanilido)-2,7-di-*tert*-butyl-9,9-dimethylxanthene),^74,84^ (b) the phenyl–nickel bond in (trispyrazolylborate)Ni(Ph)(CF_3_)_2_,^83,85^ and (c) the benzyl–copper bond in [Cu(CF_3_)_3_(CH_2_Ph)]^−^,^10,86^ which has also been discussed in the context of oxidation state ambiguities.^7,10^ Any ***OS*** assignment of the metal in these complexes necessitates a division of the two electrons in these covalent bonds, a task that is understandably complicated by their shared nature.

**Figure 8:**
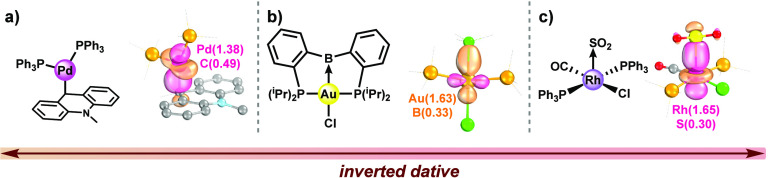
Examples of (organometallic) complexes reported by (a) Litle and Gabbai,^87^ (b) Sircoglou et al.,^88^ and (c) Muir and Ibers,^89^ with inverted dative metal–ligand bonds. Shown alongside are the σ-IBOs of relevance and their IAO charges, calculated with PBE0/def2-TZVP. Ligand side groups are hidden for clarity.

If we push past the electron-sharing bonding region in chemical space (Figure 6), we again find polarized bonds that lend themselves to simple ***OS*** assignments, even if they do sometimes violate the IUPAC ***OS*** definition, leading to the caveat where “the more electronegative atom is bonded as a Lewis acid (a so called Z-type ligand)”.^18^ Although somewhat unusual, many complexes with locally inverted dative σ-bonds (defined in Table 1) are known, and here we highlight several representative examples (Figure 8), some of which have been discussed extensively by Karen et al.,^16,18^ and include (a) the carbon–palladium bond in LPdPPh_3_ (where L is an ambiphilic phosphine-carbenium ligand),^87^ (b) the boron–gold bond in AuCl(diphosphanylborane),^88^ and (c) the sulfur–rhodium bond in RhCl(CO)(SO_2_)(PPh_3_)_2_.^89^ Despite their appeal to reasoning based on MO diagrams, Karen et al. remind us that this is “not to be taken as an instruction to start using quantum-chemical calculations to obtain [***OS***]”^18^ and remark on the “inherent degree of ambiguity because of the variety of computational methods available and of the basis-set data to choose from”.^17^ Clearly, we believe that computations can be a rich source of insight into chemical concepts, including ***OS***s, but we agree with the caution with respect to exactly how this is done.

From a delocalized perspective, the complexes shown in Figure 8 are expected to have inverted ligand fields (ILFs). However, not all complexes with ILFs have locally inverted σ-bonds. The ligand fields of complexes with electron-sharing bonds, such as (trispyrazolylborate)Ni(Ph)(CF_3_)_2_ and [Cu(CF_3_)_3_(CH_2_Ph)]^−^ (Figure 7), are classified as inverted.^7,43^ Indeed, even some complexes containing only classically dative metal–ligand bonds, such as **1**^**3+**^ (Table S14) and [Cu(CF_3_)_4_]^−^,^5^ fall under the expansive definition of ligand field inversion. From the localized perspective, the presence of an ILF is not enough to warrant an ***OS*** assignment of the metal as fully (2 e^–^) reduced, i.e., Co(I) in **1**^**3+**^ or Cu(I) in [Cu(CF_3_)_4_]^−^.

**Figure 9:**
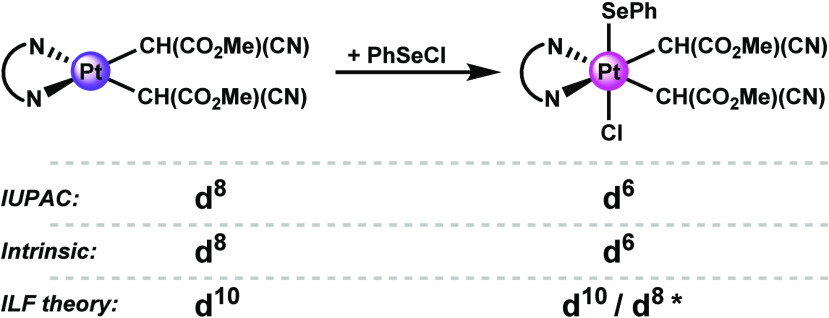
Pt d-configurations according to formal (IUPAC), localized (Intrinsic), and delocalized (ILF theory) approaches. *Different applications of ILF theory lead to different d-configurations of the product complex. See discussion in the main text for more details.

Manca and co-workers recently computationally investigated the oxidative addition of PhSeCl to a square-planar formal d^8^ Pt(II) complex (Figure 9),^90^ resulting in an octahedral formal d^6^ Pt(IV) species, as previously reported experimentally.^91^ Similarly to [Cu(CF_3_)_4_]^−^, Manca and co-workers describe the ligand field of the square-planar formal d^8^ Pt(II) reactant complex as inverted, and it is therefore said to be physically (2 e^–^) reduced, i.e., d^10^ Pt(0). Interestingly, their application^90^ of ILF theory to the octahedral product complex differs from Lancaster’s treatment of octahedral complexes of group 10 transition metals (see Supporting Information for details):^43^ for this formal d^6^ Pt(IV) species, Manca and co-workers propose a doubly inverted (4 e^–^ reduced) d^10^ Pt(0) description. Through their ligand field analysis, they conclude that the metal center “maintains the d^10^ configuration” throughout the reaction, so the electron holes created by the complex’s oxidation “are mainly centered on the ligands”.^90^ These conclusions collide with the traditional understanding of oxidative addition reactions of transition metal complexes. By contrast, the Intrinsic ***OS*** approach recovers the picture painted by the ***OS*** formalism. The intrinsic d-configuration of the square-planar reactant complex is, as expected, d^8^ Pt(II), and the product complex is intrinsically d^6^ Pt(IV) (Table S11). These results strongly indicate that the oxidation process is metal centered, although the metal–ligand σ-bonds of course adjust their polarities in response, becoming in general more electron-sharing (Δσ-gain_reaction_ = 1.35). This marks an evolving perspective on what have previously been called “essentially redox-neutral elimination[s]”,^83^ a phenomenon that has been discussed elsewhere.^7,10,14,43,83^

Intrinsic ***OS***s (defined in Table 1) minimize the impact of user choices since the IBO framework they are based on is known to show little method/basis-set dependency.^49^ They also afford a simple and rapid means to know when to apply the exception of a “reversibly-bonded Lewis-acidic electronegative ligand”^18^ in IUPAC’s 2016 ***OS*** definition, i.e., the identification of a locally inverted σ-bond. Furthermore, the changes in Intrinsic ***OS*** during reactions can be readily examined via application of electron flow analysis, a technique for which the IBOs are particularly well-suited.^e^^,^^51^ Although similar to some other approaches based on localized orbitals,^11,26^ it is less algorithmic, encouraging users to think of bonding scenarios as lying on a spectrum (Figure 6).

## Conclusions

Intrinsic oxidation states can be computed with Intrinsic Bonding Orbitals. The Intrinsic ***OS*** framework is applicable to a wide variety of transition metal complexes, spanning different points along the spectrum of covalency (classical, electron-sharing, inverted) and allows a distinction to be drawn between (delocalized) inverted ligand fields and (locally) inverted bonds. Applying the Intrinsic ***OS*** approach to a cobalt complex in five oxidation states, isolated and characterized by Hunter and co-workers,^65^ we found a smooth and continuous transition from the low- to the high-valent extremes, which both consist of expected bonding motifs. Two molecular descriptors (π-loss and σ-gain) reveal similar amounts of cumulative electron-sharing character in the σ-bonding frameworks of the Co(III) center from Hunter’s group and the much-discussed Cu(III) center in [Cu(CF_3_)_4_]^−^. While this could lead to a Co(I) reassignment, via ligand field inversion arguments, we resist this temptation and maintain that the Co(III) description is far more useful, since it agrees not only with IUPAC’s ***OS*** formalism but also with our bonding analyses and also with the experimental characterization. We propose the use of Intrinsic ***OS***s in combination with IUPAC’s definitions to identify exceptions due to inverted bonding. The ***OS*** is, like so many chemical concepts, sometimes challenging to precisely define. However, as we hope to have demonstrated here, oxidation states often conveniently track subtle changes in electronic structure and thus carry precious chemical insight.

## Data Availability

The (raw) computational data can be found at 10.34894/GNIO5G.

## Supporting Information Available

Computational details, structural parameters, intrinsic and effective ***OS*** analysis, ligand field analysis, and Cartesian coordinates of all optimized structures (PDF)

## Notes

The data in this article are based on a chapter in the Ph.D. thesis of I. F. Leach.

## Footnotes

a A CAS SciFinder search indicates 121 citations for Hoffmann et al.’s 2016 work on ILFs (ref (6), Figure S2), many of which find the presence of ILFs in a wide variety of coordination compounds. We encourage the curious reader to explore the cited literature for examples.

b Note that the IBOs in Figure 2 are of local δ-symmetry to the metal but local π-symmetry to the ligands and will thus be labeled as π-IBOs for convenience.

c This value of σ-gain (1.83 *e*) is obtained at the reported (PBE0/def2-TZVPP//B97-3c) level of theory, but recomputing at the r^2^SCAN-3c and PBE0/def2-TZVP//r^2^SCAN-3c levels used here yields almost identical results (1.82 *e*, and 1.83 *e*, Table S12).

d In their 2019 work (ref (7)), Lancaster and co-workers state that inversion applies “if the frontier orbital(s) composition [the σ-LUMO] comprises significantly less than 50% [M] 3d-character”. See the Ligand Field Analysis section of the Supporting Information for more details.

e For a representative example of electron flow analysis of a redox reaction, in which the intrinsic d-configuration of the metal changes, see ref (10).
