# Supplementary material for: Oxidation States: Intrinsically Ambiguous?
Source: ACS Cent Sci. 2024 Jun 25;10(7):1406–14. doi: 10.1021/acscentsci.4c00825 (PMC11273457; doi:10.1021/acscentsci.4c00825)
Supplement: Supplementary file 1 — oc4c00825_si_001.pdf [file oc4c00825_si_001.pdf]

Supporting information for

Oxidation States: Intrinsically Ambiguous?

Isaac F. Leach,[ab*] and Johannes E. M. N. Klein[a*]

[a]*Molecular Inorganic Chemistry, Stratingh Institute for Chemistry, University of Groningen, Nijenborgh 3, 9747 AG Groningen, The Netherlands. Email: j.e.m.n.klein@rug.nl.*

[b]*Zernike Institute for Advanced Materials, University of Groningen, Nijenborgh 3, 9747 AG Groningen, The Netherlands. Email: i.f.leach@rug.nl.*

Contents

**Computational Details**	**S1**
**Structural Parameters**	**S2**
**Effective Oxidation State Analysis**	**S2**
**Intrinsic *OS* Analysis**	**S3**
**Ligand Field Analysis**	**S11**
**Cartesian Coordinates of Optimized Geometries**	**S13**
**References**	**S20**

Computational Details

Geometries of all the cobalt complexes were optimized in the gas phase, without symmetry constraints, with the efficient composite DFT method r^{2}SCAN-3c from Grimme and co-workers[1] in ORCA 5.0.4.[2-3] Numerical frequency analyses confirmed the nature of the stationary points.

At the optimized geometries, single point calculations were performed at the PBE0[4]/def2-TZVP[5] level of theory. These calculations were used to perform IBO[6] (using *exp2*) and EOS[7] analyses as implemented in IboView v20211019. For the analysis of the open shell species (**1** and **1^{2+}**), the Quasi-Restricted Orbital (QRO) transformation was applied.[8] A conservative integration grid was used (*DefGrid2*), and the *TightSCF* keyword specified an energetic convergence criterion if 10^{-8} Hartree. The stability of all wavefunctions was verified; no broken symmetry solutions were found.

Example input line:
! r2SCAN-3c TightOpt NumFreq DefGrid2 TightSCF NoTrah

Additional single point calculations using the same settings were performed on the gold, nickel, copper, palladium, and rhodium complexes showed in Figures 7 and 8. These calculations used the previously reported geometries (Table S1).

Table S1: Geometries of the various metal complexes used in this work, their location in the main text, the level that was used for optimization and the corresponding reference.

#	*Fig. #*	Metal	Geometry optimized with:	Ref.
1	*2*	Co	r^{2}SCAN-3c[1]	*This work*
2	*7a*	Au	B97-3c[9]	[10]
3	*7b*	Ni	M06-L[11]	[12]
4	*7c*	Cu	B97-3c[9]	[13]
5	*8a*	Pd	MPW1PW91[14]/[cc-pVTZ-PP(Pd); 6-31G(d',p')(P/Cl); 6-31G(d')(C/N/O); 6-31G(H)][15-17]	[18]
6	*8b*	Au	B3PW91[19]/[SDD(Au,P),[20]6-31G(d,p)(other atoms)[21]]	[22]
7	*8c*	Rh	r^{2}SCAN-3c[1]	*This work*

Structural Parameters

Table S2: Experimental (XRD)[23] and r^2-3c (DFT) optimized key geometric parameters of **1**.

		**Co-P avg. (pm)**	**P-Co-P, trans (deg.)**		**C-C, backbone avg. (pm)**
			a	***b***	
**1$^-$**	**XRD**	211.7	128.9	128.9	133.4
	**DFT**	209.9	118.3	118.3	133.3
	**Dev.**	1.8	10.6	10.6	0.1
**1^0**	**XRD**	213.3	139.8	111.9	132.5
	**DFT**	217.4	134.2	115.2	133.4
	**Dev.**	−4.1	5.6	−3.3	−0.9
**1$^+$**	**XRD**	217.6	159.1	153.5	131.9
	**DFT**	217.1	156.5	156.3	133.0
	**Dev.**	0.5	2.7	−2.8	−1.1
**1^{2+}**	**XRD**	228.9	174.4	170.5	132.4
	**DFT**	230.2	176.1	169.6	132.9
	**Dev.**	−1.3	−1.7	0.9	−0.5
**1^{3+}**	**XRD**	235.2	180.0	180.0	132.4
	**DFT**	237.5	180.0	180.0	132.8
	**Dev.**	−2.3	0.0	0.0	−0.4

Effective Oxidation State Analysis

Each calculated Kohn−Sham wavefunction was analysed with the Effective Oxidation State (EOS) formalism from Salvador and co-workers,[7] as implemented in IboView. The EOS_{Co} generally matches the formal and intrinsic $\boldsymbol{OS}_{Co}$ (Table S3). The reliability indices (R) decrease as $\boldsymbol{OS}_{Co}$ increase. The only discrepancy between EOS_{Co} and $\boldsymbol{OS}_{Co}$ is found for **1^{3+}**, when a simple fragmentation pattern (**A**) is used *i.e.*, only two fragments were defined: the metal centre + all other atoms. The more chemically intuitive fragmentation scheme (**B**), which groups together covalently bound atoms (Co + 2 x MeCN + 2 x bis(diphenylphosphino)ethylene) is therefore recommended. For **1^{3+}**, both **A** and **B** have low reliability values, approaching 50% (where two different EOS assignments are equally probable).

We noted a very similar behaviour of EOS on the fragmentation pattern defined for $[Cu(CF_3)_4]^{1-}$,[13] where the choice of fragmentation pattern yielded either Cu(I) or Cu(III) assignments. We thus stress the importance of clearly reporting the chosen fragmentation scheme when employing this analysis.

Table S3: Effective Oxidates State of the Co centre in **1**. Two fragmentation patterns were used: **A** (Co, MeCN, 2 x bis(diphenylphosphino)ethylene) and **B** (Co, all other atoms). Calculated with PBE0/def2-TZVP//r^{2}SCAN-3c.

Complex	Fragmentation	EOS(Co), R (%)
**1⁻**	**A**	−1, 95.6
	**B**	−1, 94.6
**1⁰**	**A** (α)	0, 100.0
	(β)	0, 100.0
	**B** (α)	0, 97.7
	(β)	0, 96.9
**1⁺**	**A**	+1, 76.4
	**B**	+1, 65.1
**1²⁺**	**A** (α)	+2, 71.6
	(β)	+2, 71.6
	**B** (α)	+2, 57.9
	(β)	+2, 57.9
**1³⁺**	**A**	+3, 59.4
	**B**	+1, 57.1

Intrinsic *OS* Analysis

However, preferred reference frames have emerged–which ultimately contain the same information but offer it to us with unequal willingness as it is condensed into fewer orbitals (consider that even for modestly-sized metal complexes, like $[Cu(CF_3)_4]^-$, *N* >150, so individual analysis of all the orbitals is often untenable). Localized orbitals offer a convenient basis in which to analyze bonding, as all the scattered contributions to molecular interaction are naturally grouped together during localization.

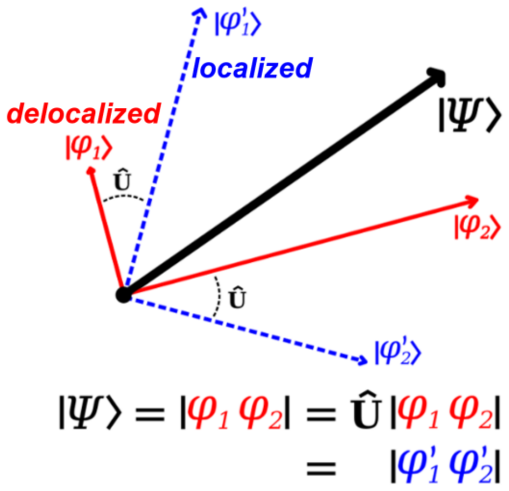

Figure S1: A wavefunction as a vector in Hilbert space, **Ψ**, being expressed by the canonical orbitals, **φ** (red), and the localized orbitals, **φ'** (blue) – related to each other *via* the unitary transformation (or *rotation*), **Û**. Adapted with permission, © Isaac F. Leach.[24]

$$\Psi = \begin{vmatrix} \varphi_1(1) & \cdots & \varphi_1(n) \\ \vdots & \ddots & \vdots \\ \varphi_n(1) & \cdots & \varphi_n(n) \end{vmatrix} = |\varphi_1 \cdots \varphi_n| = \hat{\mathrm{U}}\ |\varphi_1 \cdots \varphi_n| \ = |\varphi_1' \cdots \varphi_n'| \qquad \textbf{(eq. S1)}$$

Table S4: IBO analysis of (NON)Al-Au-P^{t}Bu$_3$ (Figure 7a), calculated *via* PBE0/def2-TZVP single point calculations at the reported (B97-3c optimized) geometry from Leach *et al*.[10] All orbitals shown are doubly occupied, and drawn with arbitrary colours to enclose 80% of their electron density.

		d-orbitals		
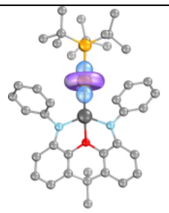	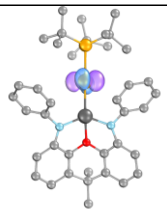	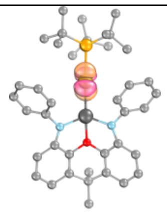	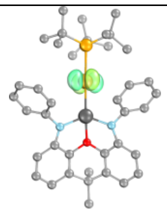	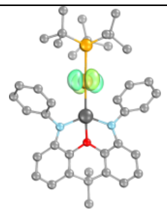
Au(1.99)	Au(2.00)	Au(1.95)	Au(2.00)	Au(1.96)
		σ-bonds		
	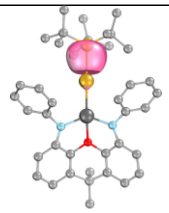		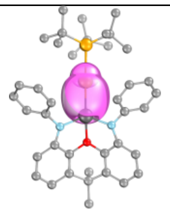	
	Au(0.18), P(1.69)		Au(0.82), Al(1.16)	
		n = 10, σ-gain = 1.00, π-loss = −0.12		

Table S5: IBO analysis of (trispyrazolylborate)Ni(Ph)(CF_3)$_2$ (Figure 7b), calculated *via* PBE0/def2-TZVP single point calculations at the reported (M06-L optimized) geometry from Steen *et al*.[12] All orbitals shown are doubly occupied, and drawn with arbitrary colours to enclose 80% of their electron density.

d-orbitals		
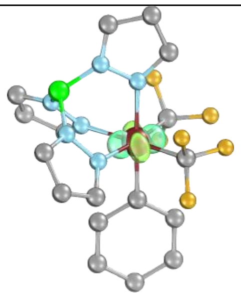	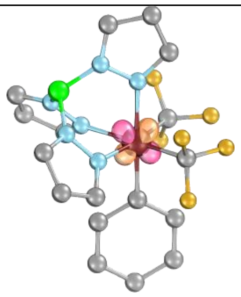	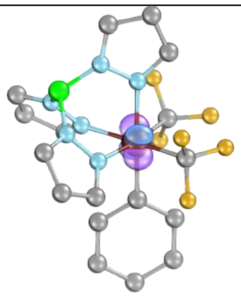
Ni(1.98)	Ni(1.98)	Ni(1.98)
σ-bonds		
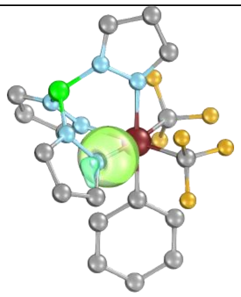	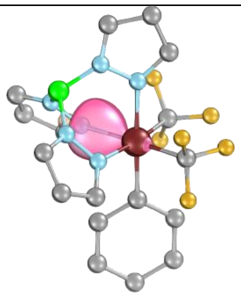	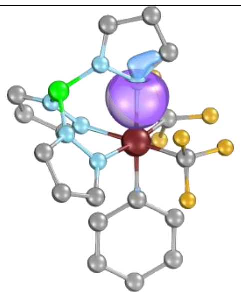
Ni(0.17), N(1.72)	Ni(0.17), N(1.72)	Ni(0.12), N(1.75)
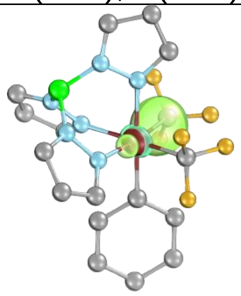	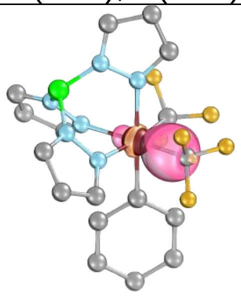	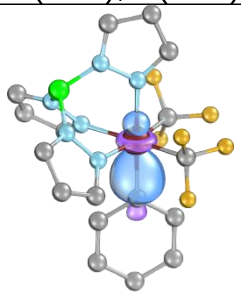
Ni(0.69), C(1.26)	Ni(0.69), C(1.26)	Ni(1.01), C(0.96)
n = 6, σ-gain = 2.84, π-loss = −0.06		

Table S6: IBO analysis of $[Cu(CF_3)_3(CH_2Ph)]^-$ (Figure 7c), calculated *via* PBE0/def2-TZVP single point calculations at the reported (B97-3c optimized) geometry from Leach *et al*.[13] All orbitals shown are doubly occupied, and drawn with arbitrary colours to enclose 80% of their electron density.

d-orbitals			
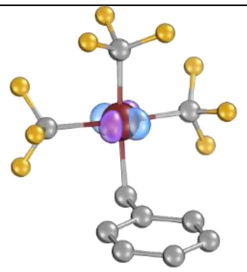	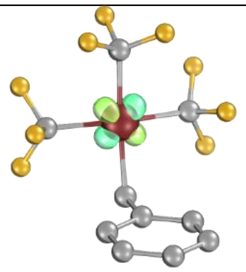	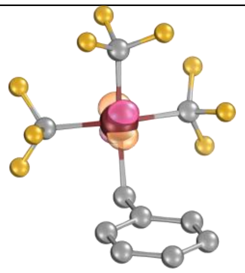	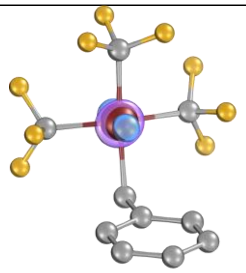
Cu(1.98)	Cu(1.98)	Cu(1.99)	Cu(2.00)
σ-bonds			
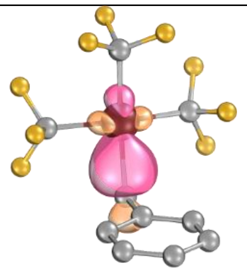	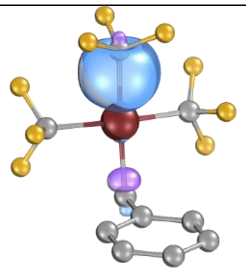	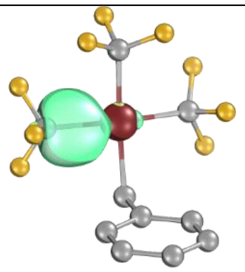	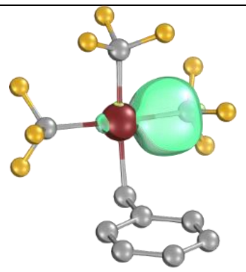
Cu(1.04), C(0.89)	Cu(0.20), C(1.57)	Cu(0.34), C(1.54)	Cu(0.34), C(1.54)
n = 8, σ-gain = 1.77, π-loss = −0.05			

Table S7: IBO analysis of $LPdPPh_3$ (Figure 8a), calculated *via* PBE0/def2-TZVP single point calculations at the reported (MPW1PW91 optimized) geometry from Litle and Gabbai.[18] All orbitals shown are doubly occupied, and drawn with arbitrary colours to enclose 80% of their electron density.

d-orbitals				
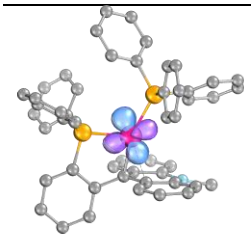	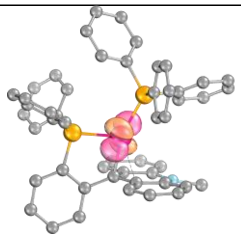	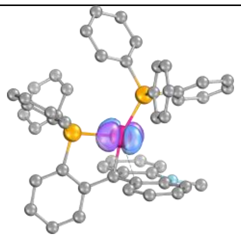	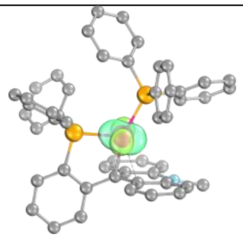	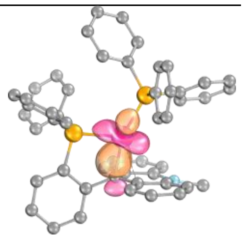
Pd(1.90)	Pd(1.95)	Pd(1.95)	Pd(1.98)	Pd(1.38), C(0.49)
σ-bonds				
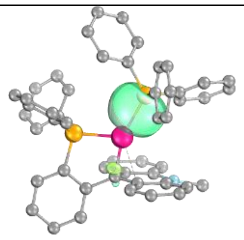		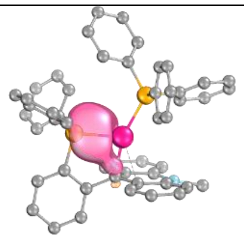		
Pd(0.20), P(1.66)		Pd(0.20), P(1.59)		
n = 10, σ-gain = 0.22,* π-loss = −0.22*				

*Note that in this case, the π-loss is calculated with respect to the four leftmost *d*-orbitals, while σ-gain includes the two σ-bonds and the rightmost *d*-orbital.

Table S8: IBO analysis of AuCl(diphosphanylborane) (Figure 8b), calculated *via* PBE0/def2-TZVP single point calculations at the reported (B3PW91 optimized) geometry from Sircoglou.[22] All orbitals shown are doubly occupied, and drawn with arbitrary colours to enclose 80% of their electron density.

d-orbitals				
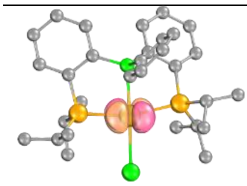	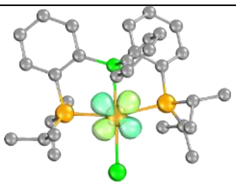	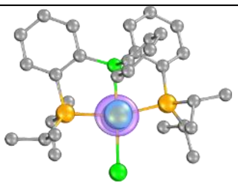	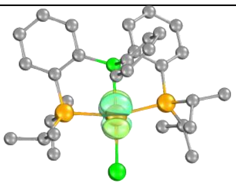	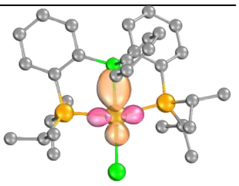
Au(1.96)	Au(1.96)	Au(1.99)	Au(1.99)	Au(1.63), B(0.33)
σ-bonds				
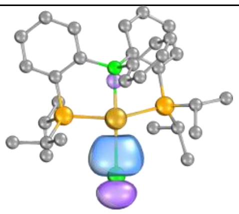	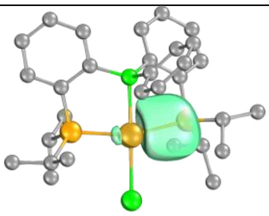	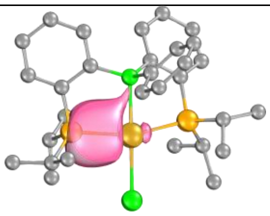		
Au(0.10), Cl(1.79)	Au(0.35), P(1.50)	Au(0.37), Al(1.47)		
n = 10, σ-gain = 0.45,* π-loss = −0.10*				

*Note that in this case, the π-loss is calculated with respect to the four leftmost *d*-orbitals, while σ-gain includes the three σ-bonds and the rightmost *d*-orbital.

Table S9: IBO analysis of $RhCl(CO)(SO_2)(PPh_3)_2$ (Figure 8c), calculated *via* PBE0/def2-TZVP single point calculations at the optimized r[2]SCAN-3c geometry. All orbitals shown are doubly occupied, and drawn with arbitrary colours to enclose 80% of their electron density.

d-orbitals			
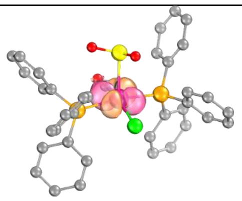	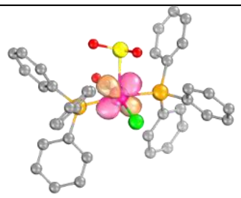	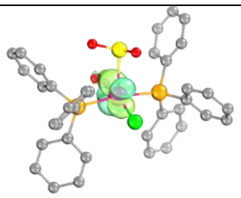	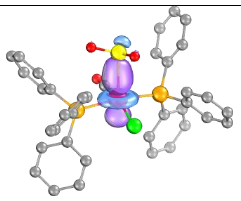
Rh(1.75)	Rh(1.92)	Rh(1.74)	Rh(1.65), S(0.30)
σ-bonds			
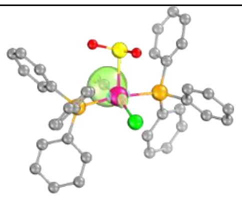	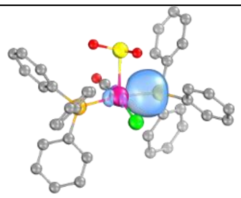	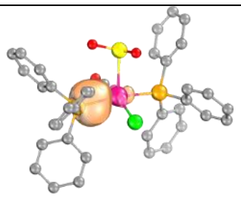	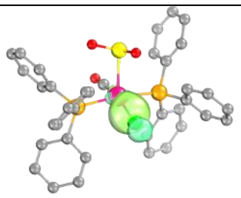
Rh(0.38), C(1.56)	Rh(0.37), P(1.51)	Rh(0.40), P(1.50)	Rh(0.28), Cl(1.66)
n = 8, *σ-gain* = 1.08, *π-loss* = −0.59*			

*Note that in this case, the π-loss is calculated with respect to the three leftmost *d*-orbitals, while σ-gain includes the four σ-bonds and the rightmost *d*-orbital.

Table S10: Intrinsic *d*-configurations (*n*) of **1** and $[Cu(CF_3)_4]^-$ with the corresponding metal orbital populations, q_{IAO}, and occupations between paratheses. Calculated with PBE0/def2-TZVP//r^{2}SCAN-3c, isosurfaces enclose 80% of the electron density of each orbital, hydrogen atoms hidden for clarity, colours are arbitrary.

Complex	*n*	*d*-orbitals				
**1^-**	**10**	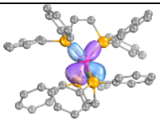 **1.62** (2)	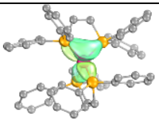 **1.62** (2)	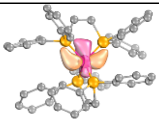 **1.75** (2)	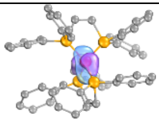 **1.75** (2)	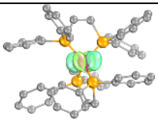 **1.82** (2)
**1^0**	**9**	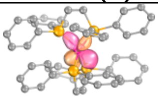 **1.72** (2)	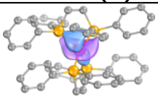 **1.74** (2)	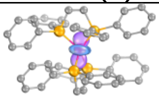 **1.85** (2)	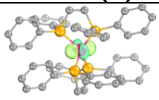 **1.90** (2)	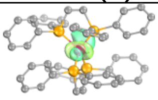 **1.68** (1)
**1^+**	**8**	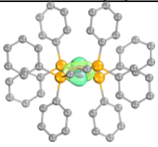 **1.82** (2)	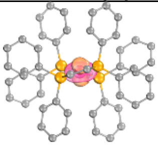 **1.82** (2)	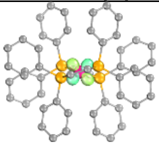 **1.89** (2)	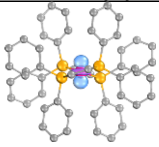 **1.92** (2)	
**1^{2+}**	**7**	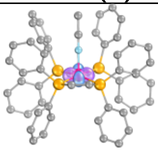 **1.90** (2)	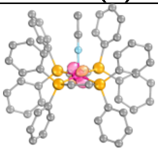 **1.93** (2)	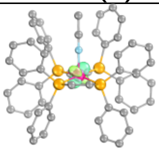 **1.94** (2)	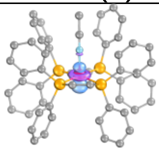 **1.78** (1)	
**1^{3+}**	**6**	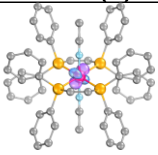 **1.93** (2)	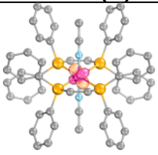 **1.93** (2)	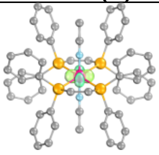 **1.94** (2)		
**$[Cu(CF_3)_4]^-$**	**8**	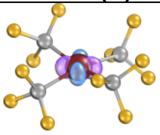 **1.97** (2)	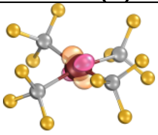 **1.98** (2)	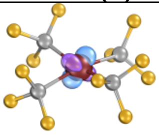 **1.98** (2)	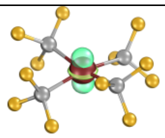 **2.00** (2)	

Table S11: IBO analysis of the oxidative addition of PhSeCl to a square-planar formal d[8] Pt(II) complex, with the corresponding orbital populations, q_{IAO}. Calculated with PBE0/def2-TZVP//r^{2}SCAN-3c. Isosurfaces enclose 80% of the electron density of each orbital, some ligand groups are hidden for clarity, colours are arbitrary.

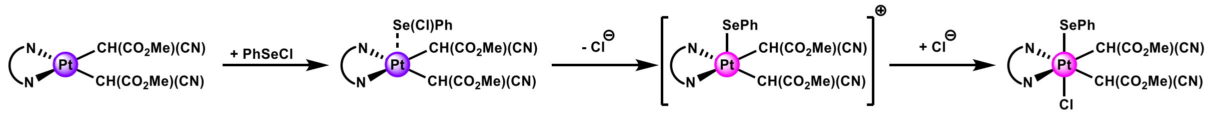

Formally: **d^8 Pt(II)** → **d^8 Pt(II)** → **d^6 Pt(IV)** → **d^6 Pt(IV)**

**Complex**	**Intrinsic *d*-config.**			**σ-bonds**		
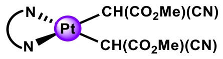	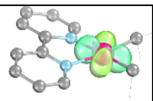 Pt(1.96)	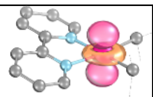 Pt(1.98)	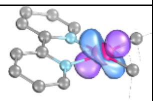 Pt(1.89)	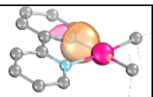 Pt(0.20) N(1.72)	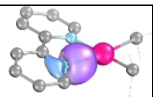 Pt(0.19) N(1.72)	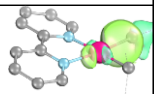 Pt(0.50) C(1.33)
	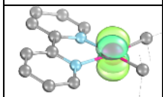 Pt(1.95)			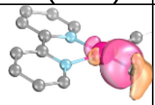 Pt(0.51) C(1.32)		
	n = 8, *σ-gain* = 1.40, *π-loss* = −0.22					
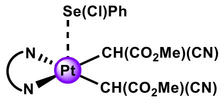	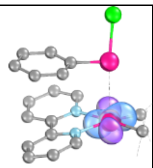 Pt(1.97)	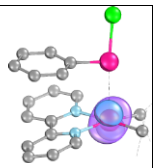 Pt(1.96)	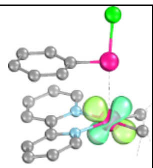 Pt(1.91)	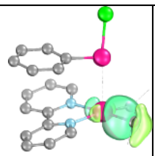 Pt(0.53) C(1.29)	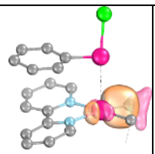 Pt(0.53) C(1.30)	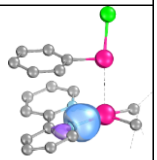 Pt(0.18) N(1.73)
	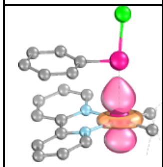 Pt(1.82)			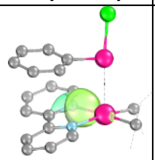 Pt(0.18) N(1.72)		
	n = 8, *σ-gain* = 1.42, *π-loss* = −0.34					
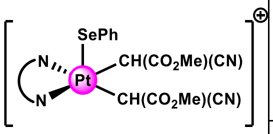	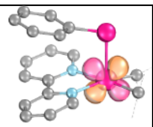 Pt(1.96)	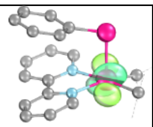 Pt(1.98)	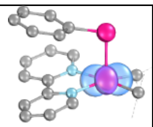 Pt(1.97)	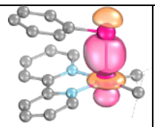 Pt(1.07) Se(0.91)	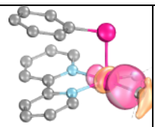 Pt(0.65) C(1.20)	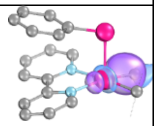 Pt(0.65) C(1.21)
				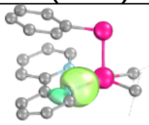 Pt(0.18) N(1.71)	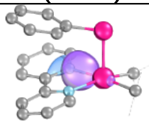 Pt(0.18) N(1.70)	
	n = 6, *σ-gain* = 2.73, *π-loss* = −0.09					

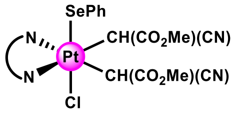	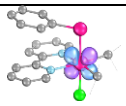 Pt(1.96)	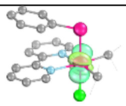 Pt(1.98)	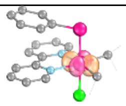 Pt(1.98)	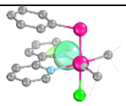 Pt(0.16) N(1.71)	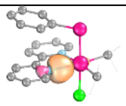 Pt(0.17) N(1.71)	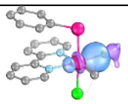 Pt(0.68) C(1.19)
				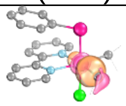 Pt(0.72) N(1.17)	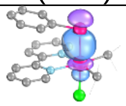 Pt(0.77) Se(1.16)	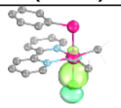 Pt(0.25) Cl(1.60)
	n = 6, *σ-gain* = 2.75, *π-loss* = −0.08					

Table S12: Intrinsic atomic orbital (IAO) partial charges of Co in **1**^{x}.

	Method	**1**$^-$	**1**^{0}	**1**$^+$	**1**$^{2+}$	**1**$^{3+}$
**IAO charge**	PBE0/def2-TZVP*	0.085	0.170	0.065	0.566	0.707
	r^{2}SCAN-3c	0.020	0.200	0.075	0.535	0.674

**via* single point calculations at the r^{2}SCAN-3c optimized geometries

Table S13: *σ-gain* and *π-loss* of the central metal ion in several TM complexes.

		**Complex**					
	**Method**	**1**$^-$	**1**^{0}	**1**$^+$	**1**$^{2+}$	**1**$^{3+}$	**[Cu(CF$_3$)$_4$]$^-$**
σ-gain	PBE0/def2-TZVP*	0.52	0.74	1.33	1.59	2.16	1.82
	r^{2}SCAN-3c	0.54	0.88	1.42	1.69	2.18	1.83
π-loss	PBE0/def2-TZVP*	−1.45	−0.94	−0.51	−0.34	−0.19	−0.06
	r^{2}SCAN-3c	−1.58	−1.15	−0.65	−0.46	−0.29	−0.08

**via* single point calculations at the r^{2}SCAN-3c optimized geometries

Ligand Field Analysis

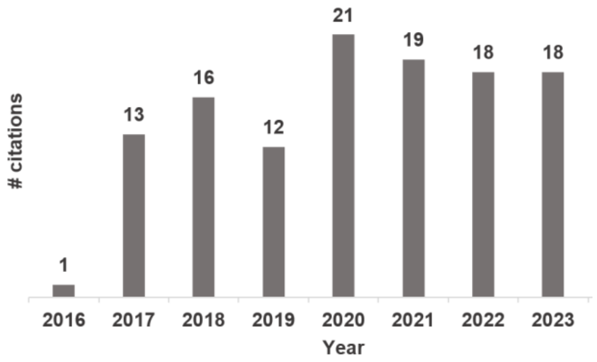

Figure S2: Citations per year, obtained *via* a CAS SciFinder search on 16/11/2023, of Hoffmann *et al.*'s 2016 work on inverted ligand fields (*Chem. Rev.* **2016**, *116*, 8173−8192, ref [25]).

In the context four-coordinate formal copper(III) complexes, Lancaster and co-workers have proposed computationally that a ligand field can be classified as inverted "if the frontier orbital(s) composition comprises significantly less than 50% [metal] 3d-character".[26-27] The character of the frontier orbital (the LUMO) is obtained *via* Löwdin population analysis. Later, this procedure was extended to six-coordinate formal d^6 nickel(IV) complexes, by averaging over the different (low-lying) virtual orbitals (of σ-symmetry).[28] The metal is argued to be in a two-electron reduced state *i.e.* d^8 nickel(II).

Manca and co-workers also extended the ILF analysis of canonical orbitals to octahedral (group 10) metal complexes.[29] Their treatment of formal d^6 platinum(IV) complexes deviates significantly from Lancaster's nickel systems. One minor difference is the use of a 60% cutoff for inversion, while a major difference is that instead of averaging over the two perpendicular inverted orbitals, they are each taken as separate instances of inversion and therefore a four-electron reduction of the metal centre is proposed *i.e.* d^{10} platinum(0).[29]

It seems therefore that the inverted ligand field model is applied to analogous cases in inconsistent ways, but here we follow Lancaster and co-workers and apply ILF analysis using a 50% cutoff and averaging the ligand contribution over perpendicular orbitals. The ligand field of the high-valent cobalt(III) complex $\mathbf{1}^{3+}$ was analyzed by examination of the Löwdin populations to the virtual orbital space (Table S13). The averaged cobalt d-orbital contribution to the σ-LUMOs (47%) falls under this definition of an inverted ligand field, similar to $[Cu(CF_3)_4]^-$ (37%, at the same PBE0/def2-TZVP//r^{2}SCAN-3c level of theory). Similar values for $[Cu(CF_3)_4]^-$ are found with different approaches.[13, 26-27]

Table S14: The canonical (delocalized) σ-LUMOs, and their metal *d*-orbital contributions (%, *via* Löwdin population analysis), calculated with PBE0/def2-TZVP//r^{2}SCAN-3c. In **1**$^{3+}$, hydrogen atoms (and the axially coordinating acetonitrile molecules in the LUMO) are hidden for clarity. Isosurfaces drawn to enclose 80% of the electron density.

Complex	Orbital #	% *d* (M)
**1**$^{3+}$	LUMO	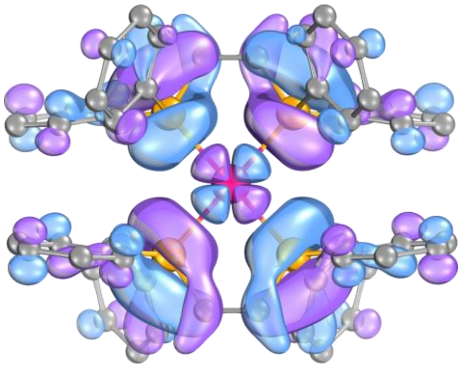 39.4 %
	LUMO+1	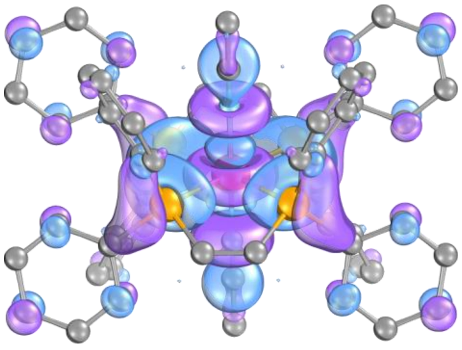 54.7 %
		Average: 47.1 %
$[Cu(CF_3)_4]^-$	LUMO	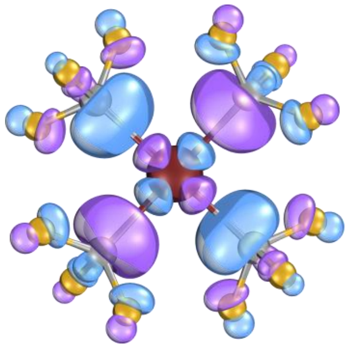 37.0 %

Cartesian Coordinates of Optimized Geometries

Complex: 1^{-}

Co	0.00016268793285	-0.01051818167145	-0.00003114701703
P	1.49016580255422	-1.14860356987439	-0.94675205178419
P	-0.45678166368319	0.89273275628110	-1.83700955923797
C	1.24400464606297	-1.01974247395594	-2.78967496726638
H	1.83810388954294	-1.61322755289440	-3.48761855155652
C	0.37538957887580	-0.09271561342508	-3.19332527940450
H	0.19902652309551	0.12465448069634	-4.24853428713340
C	1.67508618179053	-2.97841175394445	-0.75420643142965
C	0.97158670216223	-3.87301754619289	-1.56689358511685
H	0.33710213798143	-3.48721915262877	-2.36149499012335
C	1.06957965261294	-5.24636862595770	-1.36567992793358
H	0.50582838229949	-5.92372870554354	-2.00216998337314
C	1.86852016438730	-5.75295310447852	-0.34519599986523
H	1.94295803293932	-6.82600983772445	-0.18843611125933
C	2.55078193822322	-4.86976337190490	0.49043021515881
H	3.16781142681851	-5.25276477759484	1.30009204496508
C	2.44681638275967	-3.49840278509556	0.29318101705069
H	2.98941635619518	-2.81398339941257	0.94275341841791
C	3.30901204631906	-0.69015173406337	-0.96520137045233
C	3.65790257814905	0.58929258823726	-0.53451737418082
H	2.86987648248158	1.23598553591082	-0.15755867238268
C	4.98048495267977	1.01994285269441	-0.57243230459407
H	5.23140040673947	2.01459559574205	-0.21360929853032
C	5.97688517215428	0.17370585961345	-1.04918703516760
H	7.01211043339201	0.50532735346406	-1.07493349458577
C	5.63969092638113	-1.10424983649696	-1.49116262086886
H	6.41126033033770	-1.77124401973814	-1.86911989975235
C	4.31605714865768	-1.53206059721250	-1.45040730191165
H	4.06484594273592	-2.53472635732310	-1.78812459989261
C	0.16165485393791	2.57438891755984	-2.39309610286691
C	-0.08059461279704	3.10032017103925	-3.66727181330886
H	-0.70237019621775	2.54678215326082	-4.36758597117644
C	0.45032134372240	4.33063413461255	-4.04207183763535
H	0.25349687014365	4.72532852514531	-5.03623534077224
C	1.23364128101843	5.05642316799109	-3.14593699962343
H	1.64809103417495	6.01819395632503	-3.43813811098337
C	1.48091084329553	4.54243130605099	-1.87644901948034
H	2.08755800195065	5.10194573651796	-1.16871470002671
C	0.94906516069685	3.30920810629773	-1.50761872856947
H	1.13472079103394	2.88905300582303	-0.51963926783210
C	-2.17687397775528	0.95799376615970	-2.53003969278267
C	-2.95860893651017	2.11908722141663	-2.52558894909362
H	-2.50675605694133	3.06833131066069	-2.24544219494162
C	-4.30626729531800	2.07427625541132	-2.87095964795263
H	-4.89638847253736	2.98792898914209	-2.85531719204085
C	-4.90210153376549	0.86708056478948	-3.22623043711771
H	-5.95713631655836	0.82996018985200	-3.48378289311144
C	-4.13427942865365	-0.29650572007598	-3.23348821262846
H	-4.59040943568583	-1.24648446954072	-3.50172776556285
C	-2.78970738519511	-0.25074543277506	-2.88540536765937
H	-2.19854364467356	-1.16474007417858	-2.86504268761949
P	-1.49053751577497	-1.14602363858058	0.94878165466944
P	0.45784504345507	0.89542279022812	1.83542087958279
C	-1.24363861040146	-1.01475230011521	2.79141088287696
H	-1.83782429905709	-1.60686068590615	3.49045037208875
C	-0.37429065702833	-0.08770273758833	3.19344136005469
H	-0.19744112027979	0.13105077991632	4.24828465788367
C	-1.67745744874678	-2.97589875441724	0.75885204819962
C	-0.97507190116485	-3.87028191415657	1.57273132383365
H	-0.33998932492042	-3.48423748589115	2.36673291945214
C	-1.07492468364369	-5.24379375723877	1.37346797446819
H	-0.51200356834817	-5.92098795130767	2.01087292079514
C	-1.87461971932580	-5.75075401932825	0.35377205138445
H	-1.95057972438653	-6.82392907467708	0.19856358072277
C	-2.55575994562481	-4.86782426553549	-0.48306291820814
H	-3.17334447374981	-5.25116849600145	-1.29213943026691
C	-2.44995524291069	-3.49634297107829	-0.28775536401152
H	-2.99164487850275	-2.81207826568146	-0.93825779008492
C	-3.30895469626295	-0.68596134136268	0.96693524248252
C	-3.65705550601038	0.59256998796683	0.53296290232875
H	-2.86867544076233	1.23761825392262	0.15394713384078
C	-4.97930800131018	1.02430727585298	0.57028949417119
H	-5.22966551569794	2.01817101235047	0.20889235301837
C	-5.97611632881302	0.18010372086044	1.04978277375074
H	-7.01107853814879	0.51257648493852	1.07511820547203
C	-5.63969264456084	-1.09692015443125	1.49504655328226
H	-6.41159860766313	-1.76231609394552	1.87512749892412
C	-4.31640637189955	-1.52583937564937	1.45481459713444
H	-4.06577963501216	-2.52781781961629	1.79501449945775
C	-0.15991979882030	2.57804990378894	2.38930566569524
C	0.08313774410952	3.10583206202825	3.66256615891549

Energies (au)

E(PBE0/def2-TZVP//r^{2}SCAN-3c)
= −4753.879121183299

Thermal correction*
= 0.71859757

*** 1 atm, 298K, 1M, r^{2}SCAN-3c**

H	0.70520870158498	2.55321994334740	4.36334872643315
C	-0.44735073550726	4.33679051903512	4.03584421599376
H	-0.24989218196452	4.73291696599331	5.02931177098990
C	-1.23105874295275	5.06138423126881	3.13907946650242
H	-1.64518217917656	6.02365604799309	3.43009155685917
C	-1.47913963251463	4.54554854774839	1.87049984208068
H	-2.08610948199026	5.10411098309445	1.16229093557335
C	-0.94770952122614	3.31169074224016	1.50318628211669
H	-1.13403863421886	2.89012695740803	0.51593480684972
C	2.17819351364337	0.96124166601950	2.52771398779411
C	2.96025662601655	2.12210478144958	2.52104041971651
H	2.50855452786263	3.07101745072976	2.23952394550046
C	4.30802518008700	2.07749596548403	2.86599276237052
H	4.89838758460502	2.99096215322047	2.84864489793585
C	4.90366511475114	0.87071911853038	3.22301229406141
H	5.95878771126679	0.83372971835821	3.48022498859772
C	4.13553164374278	-0.29264002332243	3.23245079792478
H	4.59150729159758	-1.24230799615713	3.50204810564316
C	2.79083614387256	-0.24707850399288	2.88481740945864
H	2.19941935183961	-1.16094524478407	2.86618366769718

Complex: 1^0

Co	0.20671696932080	-0.26929089146721	0.06368550581295
P	-1.32689823710835	-0.46930621007121	1.60410898197947
P	1.38241413162128	1.01825015715188	1.36181878414251
P	1.10884776291672	-1.63381138945012	-1.35061952737175
P	-0.77628914379994	0.73987949534583	-1.59707868491712
C	-0.48031420957573	0.09236540298189	3.15321278225870
H	-0.92548600898737	-0.11599993274886	4.12729962496366
C	0.67236135621420	0.75891320547130	3.04797964471986
H	1.21722112086561	1.09911670194595	3.93056572922229
C	-2.09588504138171	-2.04610518312838	2.20045866718638
C	-1.84192340623002	-3.21355068199225	1.48172581773000
H	-1.21161699893691	-3.16291132473229	0.59722875589111
C	-2.38507563195936	-4.42936141853638	1.89264010717515
H	-2.17514521162075	-5.32833403597639	1.31987449897531
C	-3.18531903693827	-4.48470173384746	3.02805071864363
H	-3.60964729422994	-5.43136084441098	3.35135415542773
C	-3.44648137226960	-3.32139027567876	3.75187238517903
H	-4.07601103657344	-3.36077380809024	4.63674298712021
C	-2.90739190405026	-2.10897635027697	3.33951552353456
H	-3.13573442118800	-1.20209703412858	3.89482493573423
C	-2.79603782724069	0.65196046244385	1.61685227478957
C	-4.07490719298214	0.18947003317348	1.29419315847878
H	-4.23907891907910	-0.86768871201463	1.11100658305424
C	-5.14073857007131	1.07740736046280	1.19489325444086
H	-6.12443511480280	0.70323583327706	0.92574905369962
C	-4.94979037905274	2.43551847084930	1.43135116672816
H	-5.78494606640481	3.12616499738346	1.35713569866972
C	-3.68069284887383	2.90274012738718	1.76230223360047
H	-3.51971614913022	3.96088152416720	1.95226576715898
C	-2.60981450703865	2.01866186591587	1.84584129155610
H	-1.61765701509484	2.39504863154245	2.08217549134217
C	3.14588845323136	0.59073072582333	1.75031643008676
C	4.19748385468099	1.50736519745513	1.80796291053147
H	4.00395838352179	2.56733416282153	1.67049727606523
C	5.50054149280454	1.06852754242997	2.03716689600422
H	6.31118410486845	1.79157962136602	2.07233913974325
C	5.76498131468573	-0.28357117535664	2.22677835327112
H	6.78209858448406	-0.62239718603724	2.40208032169058
C	4.71791415939210	-1.20222634810906	2.18749776184352
H	4.91562503413555	-2.26092805070480	2.32996963115116
C	3.42255705936397	-0.76844534829594	1.94192843703841
H	2.61010303460712	-1.49120920367705	1.87728021597821
C	1.45832541712749	2.84596490605009	1.20932309748861
C	0.93116952501558	3.71397060577133	2.16921706748097
H	0.47367755137695	3.31525436672536	3.07044844903183
C	0.99329539865842	5.09334546424474	1.98442848307301
H	0.57852394430945	5.75561776768261	2.73952366801055
C	1.59501630266512	5.62117077412176	0.84762131655552
H	1.64808611427096	6.69702373686211	0.70660989609057
C	2.12056965402040	4.76260758039697	-0.11555047556004
H	2.57340784019261	5.16518536835601	-1.01662190693094
C	2.03867448320236	3.38780480839431	0.05554912868347
H	2.42331479271343	2.72429144804064	-0.71581647025199
C	0.60380196672784	-1.11959593449908	-3.04478761268110
H	0.96628258342192	-1.67591415287162	-3.91043126446735
C	-0.18253104139925	-0.05029764833449	-3.15732295949883
H	-0.53234799568820	0.31546410396994	-4.12377266626174
C	2.94285939976414	-1.76573040765585	-1.62823654098289
C	3.70159175274795	-0.61864508362572	-1.38614738463964
H	3.20072070240300	0.26973311511276	-1.00871421176647
C	5.07488366586533	-0.61543667711968	-1.59829338654236

Energies (au)

E(PBE0/def2-TZVP//r^{2}SCAN-3c) = -4753.842093699805

Thermal correction* = 0.72100184

*** 1 atm, 298K, 1M, r^{2}SCAN-3c**

H	5.65012250393088	0.28197555786932	-1.38874510005114
C	5.70991916411459	-1.76799386169986	-2.05254026879733
H	6.78540511920117	-1.77428816676621	-2.20686006407472
C	4.96145141593660	-2.91389823406865	-2.30760816908049
H	5.45083159051865	-3.81407384571082	-2.66969527219338
C	3.58431670025705	-2.91391376542377	-2.10197590275253
H	3.01089860478684	-3.81368131018275	-2.30637953509870
C	0.60803280922645	-3.40609989180249	-1.32791425673370
C	1.16065584898692	-4.26740159300169	-0.36975191224616
H	1.97217621488952	-3.91766354855253	0.26507045991217
C	0.68102885357311	-5.56215880022890	-0.21915913455480
H	1.12295205150002	-6.21564785496583	0.52806161126449
C	-0.36730256474550	-6.02046818247625	-1.01595128243403
H	-0.74236847628926	-7.03306890405112	-0.89706900145075
C	-0.92840902260158	-5.17108689447637	-1.96507559494419
H	-1.74500994918591	-5.51869499224138	-2.59200604385315
C	-0.44474690071316	-3.87502574403050	-2.12206824698800
H	-0.89750625833913	-3.21859799792913	-2.86100206683759
C	-0.61032946338252	2.52750218287257	-1.99949393756463
C	-1.26684892734267	3.47001964602364	-1.19834234750641
H	-1.90022417981868	3.13337671720442	-0.38173287472334
C	-1.12462663727025	4.82912669580696	-1.44332536918125
H	-1.64533634557428	5.54540529416348	-0.81374066426044
C	-0.30760973930552	5.27338583921448	-2.48051919179032
H	-0.19499412747089	6.33717872511832	-2.67059072964877
C	0.37673011448484	4.34682294242269	-3.25953627355997
H	1.02766023223147	4.68384163043644	-4.06196031007184
C	0.23023413106316	2.98270479382476	-3.01967599719928
H	0.77957234089836	2.27179612213517	-3.63121749753447
C	-2.58525903345811	0.41649978303259	-1.86974370992465
C	-3.52005899105828	1.36579944547572	-2.28363102434645
H	-3.21649744890311	2.39334245062997	-2.45800926662520
C	-4.85183977470237	1.00259905397736	-2.47551495377132
H	-5.57198440198521	1.75517019365038	-2.78505530947351
C	-5.25883611762255	-0.31247496873044	-2.28043003834243
H	-6.29714584982714	-0.59201953455257	-2.43702373560606
C	-4.32707110787664	-1.26904893448225	-1.88310975050510
H	-4.63306994853844	-2.30004149822771	-1.72513485367063
C	-3.00402773275450	-0.90484991507632	-1.67015853215168
H	-2.28181607682286	-1.64481110456695	-1.32850518888997

Complex: 1^+				Energies (au)
Co	0.01662509852779	0.00306559539586	0.07237896223468	**E(PBE0/def2-TZVP//r^{2}SCAN -3c)**
P	1.48668579666206	0.20894339686638	1.64159351479526	**= −4753.695706542452**
P	-1.40321077689515	-0.39092152377958	1.65241265222025	
P	-1.38690179400784	0.68386470437586	-1.45186489134227	**Thermal correction***
P	1.36811100281799	-0.48472348850422	-1.57019468420376	**= 0.72611873**
C	0.71832719980347	-0.04621787465421	3.28964275047348	
H	1.32291682947962	0.02046568873602	4.19416056301016	*** 1 atm, 298K, 1M, r^{2}SCAN-3c**
C	-0.58152336402550	-0.33246345925087	3.29339517297564	
H	-1.15715389977837	-0.50629676832003	4.20251676098890	
C	1.96320809106147	1.97660286278051	1.68028964018768	
C	1.18836838505283	2.88173252285248	2.41710027250700	
H	0.37327419977776	2.52229682479480	3.03929542834228	
C	1.45883273723282	4.24434441237312	2.35865226505738	
H	0.85851854636415	4.93594525430643	2.94257148201945	
C	2.49714842435211	4.71856598273852	1.56132864505588	
H	2.70987354976448	5.78287037328773	1.51976514644810	
C	3.26797955765582	3.82296630814277	0.82456773124218	
H	4.08231180520972	4.18346529657015	0.20291873771480	
C	3.00540257239768	2.45908601673112	0.87958562875722	
H	3.61667857105340	1.77357522722642	0.30233574907684	
C	3.02608975097090	-0.76791520000727	1.81867012072141	
C	2.95792439361290	-2.12483670071496	1.48524872817881	
H	2.03003000212555	-2.54427527094013	1.10473366619473	
C	4.07928179133161	-2.93585224507583	1.61571278640272	
H	4.01839587844152	-3.98620667450506	1.34629197363857	
C	5.27679320145738	-2.39640903902286	2.07880299677583	
H	6.15666041847034	-3.02606176185530	2.17299291209312	
C	5.34296398977456	-1.05091928471833	2.43092522716901	
H	6.27243252767821	-0.63150182069611	2.80464941806316	
C	4.22119193251344	-0.23666590334526	2.30908185112923	
H	4.28328800447859	0.81111171710262	2.58755792807376	
C	-1.89503843657113	-2.14878860644930	1.50209471697319	
C	-1.09436699403512	-3.13640866105025	2.09082208425051	
H	-0.24955196684465	-2.85507248099626	2.71360522966746	
C	-1.37718623046011	-4.48205820483902	1.88477531950608	
H	-0.75624571555891	-5.23899201515463	2.35504424020776	
C	-2.45351822679276	-4.85678151233626	1.08499585967988	
H	-2.67517890788544	-5.90830476842793	0.92729632580614	
C	-3.25098787462481	-3.87896942270606	0.49619168605829	
H	-4.09549769606880	-4.16147090034937	-0.12551617584695	
C	-2.97703769757937	-2.53156813677979	0.70040875550662	

H	-3.61161726609612	-1.78246338867792	0.23886847731954	
C	-2.92592910515742	0.56824813865603	1.99361876535001	
C	-4.10669798689863	-0.00721332094660	2.46897198659915	
H	-4.16813355564889	-1.07892739812425	2.63327664267594	
C	-5.21502577574704	0.79498467137105	2.72217363590471	
H	-6.13394839992747	0.34244821122574	3.08314613303722	
C	-5.14856322307431	2.17076998130030	2.51792759652328	
H	-6.01798655840807	2.79116112457939	2.71469277692047	
C	-3.96390107579652	2.75124993573960	2.07181816928360	
H	-3.90254990081321	3.82464136466448	1.91814708732768	
C	-2.85640658359749	1.95313605830056	1.80977300656096	
H	-1.93929935665035	2.40650829451598	1.44221286308865	
C	-0.65738594060266	0.43660830719451	-3.11976669986356	
H	-1.23119430983562	0.70191766386020	-4.00785572316104	
C	0.57950059499537	-0.04849354966849	-3.17042446882028	
H	1.12043219328567	-0.20757209525602	-4.10337612481830	
C	-3.05673196721914	-0.03094152347813	-1.69915405921759	
C	-3.16187340152018	-1.21353575633807	-2.44146171569373	
H	-2.27897205328546	-1.64624134647345	-2.90639225737044	
C	-4.39022697791291	-1.84986581342561	-2.57839859499151	
H	-4.46106579878664	-2.76209487789556	-3.16341801791971	
C	-5.52334654154432	-1.32008505083833	-1.96641741196968	
H	-6.48313344697179	-1.81684094023703	-2.07344393965061	
C	-5.42380064636728	-0.14690340315249	-1.22320280707688	
H	-6.30327440511837	0.27348812739789	-0.74451379991611	
C	-4.19845839690973	0.49830527088530	-1.08857927335142	
H	-4.13609030494087	1.41135103252764	-0.50497223217078	
C	-1.61934240441292	2.50547877404361	-1.44773489631624	
C	-2.63101192036969	3.12977828281060	-2.18366839862070	
H	-3.35404440449651	2.53677301218957	-2.73673609839904	
C	-2.71837339818552	4.51791328756786	-2.20370433611552	
H	-3.50895945464323	4.99819903261650	-2.77258093978688	
C	-1.79612310688837	5.28924524728289	-1.50104830650176	
H	-1.86984939169368	6.37268568897896	-1.51967509417667	
C	-0.77686397947031	4.67117853408744	-0.78171110319038	
H	-0.04901713232748	5.26413019812331	-0.23587942845327	
C	-0.68659205146959	3.28389016208610	-0.75570153275758	
H	0.10785765131614	2.79806728586107	-0.19111348192701	
C	3.02753880398990	0.26244550784805	-1.79170210305844	
C	4.18929874420389	-0.32641018822056	-1.28209881508298	
H	4.14763071055428	-1.29949621008358	-0.80271572283895	
C	5.40882403567024	0.33577728299214	-1.38380746725608	
H	6.30418902576654	-0.13123125177305	-0.98437027430475	
C	5.48238653426880	1.58551801774781	-1.99318732780177	
H	6.43748972921831	2.09594030241929	-2.07470179088228	
C	4.32924975293858	2.17472207981585	-2.50516702559683	
H	4.37978259082898	3.14679204648390	-2.98678539676541	
C	3.10698130095489	1.52083433924100	-2.40078321050676	
H	2.20899612363886	1.99825923576956	-2.78624539482133	
C	1.60183964535710	-2.29311773160074	-1.78465291725523	
C	2.59138042045235	-2.82567319645229	-2.61642917420366	
H	3.29794519947306	-2.17078452307554	-3.11868908899194	
C	2.67758338901849	-4.20209396379161	-2.79818371333966	
H	3.45081927570213	-4.61148821757103	-3.44148817505966	
C	1.77581895881362	-5.05179250472080	-2.16253618013217	
H	1.84834854902360	-6.12564918297626	-2.30803060286255	
C	0.77809277650268	-4.52346616321411	-1.34764861917563	
H	0.06584830934620	-5.17747224272785	-0.85331617355783	
C	0.68947557761797	-3.14865618817596	-1.15957971795280	
H	-0.08837124959008	-2.73251822409078	-0.52092048121822	

Complex: 1^{2+}				Energies (au)
Co	-0.03233894482597	0.07791116612946	0.00038142855091	**E(PBE0/def2-TZVP//r^{2}SCAN-3c) = −4886.092409953781**<br><br>**Thermal correction* = 0.76998873**<br><br>*** 1 atm, 298K, 1M, r^{2}SCAN-3c**
P	1.56198470684261	-0.83646648028599	-1.41279380844049	
P	-1.50530312369711	-1.01933433575940	-1.38544550283912	
P	-1.66319398391959	1.26374259688994	1.09502441464381	
P	1.40830928493277	1.30398360504100	1.29984459546346	
N	-0.04918433574128	-1.41726191350231	1.31241207639685	
C	0.69266389087322	-1.22967321873004	-2.97593506344578	
H	1.27883841143043	-1.40110433217157	-3.87870287765270	
C	-0.63307553750315	-1.32909612761381	-2.95884160665964	
H	-1.21715671273069	-1.60362380031265	-3.83739178248562	
C	2.98695466828420	0.11676942763788	-2.05338753599596	
C	2.77290571998450	1.03079529766132	-3.09259492280400	
H	1.77719339533454	1.16922123789560	-3.50997646013856	
C	3.83674130929446	1.75639470631641	-3.61313095345868	
H	3.66770236450999	2.44952452671775	-4.43166003188863	
C	5.11766338699682	1.58511709683001	-3.09453887492459	
H	5.94927327954043	2.15009901124562	-3.50415086085447	
C	5.33478503642308	0.67131231659573	-2.06831515529125	
H	6.33786895659290	0.51577926382401	-1.68252502855664	
C	4.27635699923272	-0.06909670087438	-1.55087816452444	
H	4.46323020620670	-0.80682334043168	-0.77652436332274	

C	2.28175231616076	-2.44560116640299	-0.90326401117886
C	2.80602651684512	-2.60154123788753	0.38361468666733
H	2.76972618697163	-1.78555208054943	1.09649348597965
C	3.39514286188790	-3.80539961996562	0.75708163892589
H	3.81593906422742	-3.90736349780758	1.75340607627463
C	3.45566900462189	-4.86581993141796	-0.14204954635723
H	3.91699115927071	-5.80433815729639	0.15019854163290
C	2.93536958784027	-4.71500708621801	-1.42395874410089
H	2.99147684094566	-5.53328970242984	-2.13536779039550
C	2.35487326519270	-3.51103117835902	-1.80763378837781
H	1.96875000398659	-3.41340423577942	-2.81742591506995
C	-1.95303992849544	-2.69386521548375	-0.80030685675966
C	-1.27410946363011	-3.81646556674751	-1.28336356463390
H	-0.51569891097908	-3.71585011706260	-2.05251850455326
C	-1.57631494221247	-5.08286350507546	-0.78975928823665
H	-1.05679883026339	-5.94980475287776	-1.18687209355032
C	-2.54974498696096	-5.23835796211064	0.19165605937450
H	-2.79458608068883	-6.22880319272197	0.56403474256465
C	-3.21880265449047	-4.11994657763230	0.68536571521382
H	-3.98632057686981	-4.23229525873117	1.44571781427584
C	-2.92048642236048	-2.85242300982647	0.19998357427343
H	-3.45410015231691	-1.99426431895202	0.59315451803853
C	-3.04654759753914	-0.23812898988818	-1.96983713613736
C	-4.25087105533807	-0.93617751358596	-2.10010180082247
H	-4.31836674493602	-1.98297005374541	-1.82292346893663
C	-5.37600195309568	-0.28034300697420	-2.58902035893110
H	-6.31013813177931	-0.82445885495110	-2.69059223430835
C	-5.30777824386616	1.06232986961701	-2.95216047690745
H	-6.19198726164086	1.56821009361653	-3.32791915704871
C	-4.10150277218173	1.75075935936766	-2.85121486908587
H	-4.03714326276776	2.79304915394170	-3.14855499610114
C	-2.97310956181261	1.10019326620333	-2.36781065789861
H	-2.03396413071774	1.64041807103013	-2.29914125439077
C	-0.90212359362414	1.86955464740185	2.63930793508185
H	-1.54939636188110	2.27389034071762	3.41814161645613
C	0.42395037372891	1.92630609475606	2.70812622031711
H	0.94461059334266	2.36935785936380	3.55688896094513
C	-3.12993500977408	0.35891248360370	1.68598644370605
C	-3.01359030112033	-0.42129511903735	2.84322387825562
H	-2.07533522508707	-0.44703101581062	3.39206428856753
C	-4.10347670866887	-1.14891397778645	3.30680972221153
H	-4.01475222301569	-1.73488372267496	4.21673387431581
C	-5.31310280379097	-1.10967710382490	2.61684936005609
H	-6.16725394043425	-1.66969819434865	2.98562114214282
C	-5.43219046385225	-0.33623449899494	1.46519560553625
H	-6.37626122276821	-0.29482440020841	0.93047821343646
C	-4.34668544802634	0.39575349450550	0.99546922094545
H	-4.45489387338450	1.00162362664815	0.10132720713203
C	-2.28373867237418	2.84050157985126	0.40752505192034
C	-3.43276735915101	3.45190275227471	0.92202780038027
H	-4.02524923198317	2.96260568943780	1.68901298343596
C	-3.82174150538458	4.69997805625955	0.44871425437650
H	-4.71570427164683	5.16920689815532	0.84773340401466
C	-3.06677322704253	5.35007550028702	-0.52376118712560
H	-3.37536635528305	6.32528960556271	-0.88790877020585
C	-1.90896551973524	4.75583002010084	-1.01851274758485
H	-1.30787628529363	5.26686199946797	-1.76449961202673
C	-1.51875856517457	3.50707649667482	-0.55222202398077
H	-0.60955458010109	3.05167901072263	-0.93292030542238
C	2.19424474421542	2.81041755304818	0.63869704101266
C	2.03531195745209	3.16073282768611	-0.70067047571184
H	1.48534749242711	2.50594322093687	-1.36866953188788
C	2.60586799788600	4.32957240989407	-1.19325354188274
H	2.48930754648715	4.58891383457797	-2.24113353690160
C	3.33050561575535	5.15784184393117	-0.34385927723434
H	3.77425624737750	6.07271957342295	-0.72453587491134
C	3.49433505934724	4.81258482702835	0.99689466441100
H	4.06355655535458	5.45714930273438	1.65940926746116
C	2.93405290332425	3.64245669766543	1.49034938002752
H	3.08454418612670	3.37525689633531	2.53295272971442
C	2.75634687903606	0.43544878859861	2.17431550511700
C	4.08252732647085	0.54716446939993	1.74657361190314
H	4.33346218813447	1.19689436525128	0.91400367729749
C	5.08561305315687	-0.16065099133815	2.40067210633979
H	6.11541482487856	-0.06367738334209	2.07013567284016
C	4.77617795546129	-0.97342867111144	3.48723482248905
H	5.56396820530477	-1.51433996813767	4.00283692330801
C	3.45890287769032	-1.06897144740732	3.93173461270926
H	3.22171079364263	-1.67367999600169	4.80217445330939
C	2.45005560158487	-0.37036017103038	3.27859751498713
H	1.42674023441659	-0.43755760075645	3.63889853799029
C	-0.05464885536969	-2.41538170126284	1.89016678539790
C	-0.04277818907725	-3.67799302245852	2.58349440341053
H	-0.94852881906082	-3.78595700552910	3.18761535360233
H	0.83887457966264	-3.74154842262533	3.22880808929996
H	-0.00929136132852	-4.48726226941352	1.84656253400374

Complex: 1^{3+}				Energies (au)
Co	-0.00018257053727	-0.00001102053811	0.00000324804456	**E(PBE0/def2-TZVP//r^{2}SCAN-3c)**
P	1.57107555554019	0.95888338773757	-1.50144973328113	**= −5018.359002716051**
N	-0.00020568118300	-1.57659188070345	-0.98245242555541	
C	0.66378170667421	1.16709108113202	-3.06728771413881	**Thermal correction***
H	1.24608190470281	1.31404959989278	-3.97769491884570	**= 0.81651818**
C	2.15990413166467	2.63802102335758	-1.10056946785571	
C	1.83573077072690	3.71534522393091	-1.93447453702281	*** 1 atm, 298K, 1M, r^{2}SCAN-3c**
H	1.22878295228017	3.57092891947689	-2.82153735687411	
C	2.31500610344641	4.98907074795687	-1.64529644212227	
H	2.07545380478420	5.81441239602805	-2.30872470511559	
C	3.11228559115191	5.20146801314043	-0.52511361236230	
H	3.49468132534297	6.19497244570556	-0.31079050450190	
C	3.42896376901396	4.13385899463405	0.31239589095268	
H	4.06023855001820	4.28997231277705	1.18233883628424	
C	2.95308574905543	2.85830660122768	0.03308808661712	
H	3.20666673398043	2.04179299650458	0.69927610561903	
C	3.00797836114630	-0.00562418209483	-2.07057615152512	
C	4.31353387164598	0.27799191475818	-1.65733957060886	
H	4.51715153864445	1.10129024344354	-0.98163526717010	
C	5.37289014162945	-0.47895863656523	-2.14861213733535	
H	6.38704289707058	-0.24431421745191	-1.83990249821798	
C	5.14202494264829	-1.50927844762178	-3.05537577801059	
H	5.97570800912156	-2.08345551647766	-3.44833562018518	
C	3.84410504239158	-1.78107472460890	-3.48543649520604	
H	3.66689519116442	-2.55903153739177	-4.22211717649672	
C	2.77843439473648	-1.03943894433158	-2.99259362559338	
H	1.77241542998094	-1.24703510773847	-3.34879432791720	
C	0.00096863108905	-2.64142361315000	-1.42258924367129	
C	-0.00013964021817	-3.98230492318821	-1.94050525020468	
H	-0.89750549482751	-4.15090832981783	-2.54446727526796	
H	0.00145706717073	-4.68489973367632	-1.09911848089276	
H	0.89460201274833	-4.15144357015120	-2.54818018496107	
P	-1.57131848429005	0.95896087886990	-1.50132790700752	
C	-0.66411351669554	1.16719671082569	-3.06721683472366	
H	-1.24650243731564	1.31427409395431	-3.97754891068644	
C	-2.15990866974485	2.63812092194452	-1.10025597862303	
C	-1.83474779022103	3.71562524858020	-1.93354773109090	
H	-1.22734262949837	3.57126718374198	-2.82030519073774	
C	-2.31361906093770	4.98945571133877	-1.64416191594524	
H	-2.07332480627656	5.81493941323005	-2.30714531087919	
C	-3.11143804049560	5.20178504683943	-0.52435068525980	
H	-3.49351835325327	6.19537595883362	-0.30986556768644	
C	-3.42905428889647	4.13399784460351	0.31257739654967	
H	-4.06075049426722	4.29006152872541	1.18222426227867	
C	-2.95360511420902	2.85833828365984	0.03305099013357	
H	-3.20788926319775	2.04171940459310	0.69883308038305	
C	-3.00817643325087	-0.00559004903732	-2.07046732830336	
C	-4.31383299162848	0.27816854399163	-1.65765900714976	
H	-4.51762771678283	1.10166451635497	-0.98225695365364	
C	-5.37307353063818	-0.47889924937353	-2.14901261904572	
H	-6.38730472163865	-0.24414046872292	-1.84064885179859	
C	-5.14198379368475	-1.50947650576666	-3.05542477902206	
H	-5.97557300042821	-2.08375114387812	-3.44844100413052	
C	-3.84395217853789	-1.78142049329489	-3.48505836535661	
H	-3.66656040520106	-2.55957843326845	-4.22148344191527	
C	-2.77840139654528	-1.03967857052428	-2.99212385628917	
H	-1.77228663345018	-1.24739639003992	-3.34798259508787	
P	1.57102030120844	-0.95893175501180	1.50149528637329	
N	-0.00021545413135	1.57657142993803	0.98245580120230	
C	0.66367861357651	-1.16715575407723	3.06730292871074	
H	1.24595116890475	-1.31414462516364	3.97772323456204	
C	2.15984170619267	-2.63807144786676	1.10061511979294	
C	1.83569017440915	-3.71538589986417	1.93454067427381	
H	1.22877302007637	-3.57095809311496	2.82162328491334	
C	2.31494874636998	-4.98911617877392	1.64535755685724	
H	2.07541543889091	-5.81445046770879	2.30880217804894	
C	3.11218503395852	-5.20152867076218	0.52514653974065	
H	3.49456632654614	-6.19503712195130	0.31081743430834	
C	3.42883404363485	-4.13393016523901	-0.31238750104918	
H	4.06007162879841	-4.29005719515480	-1.18235430936404	
C	2.95297319788826	-2.85837296505936	-0.03307322257967	
H	3.20652124217299	-2.04187013682931	-0.69928555859809	
C	3.00790449765228	0.00556910386146	2.07067856437332	
C	4.31348721859723	-0.27811384332613	1.65757332144003	
H	4.51714085795807	-1.10148037761134	0.98196437969551	
C	5.37282191641621	0.47885533196260	2.14886523316984	
H	6.38699507010262	0.24415481145459	1.84026572696421	
C	5.14190565073023	1.50926779001410	3.05551024310325	
H	5.97556911174857	2.08346664208845	3.44847880540995	
C	3.84395673495922	1.78113628586313	3.48543757946052	

H	3.66670507497030	2.55916868517463	4.22202838102604
C	2.77830873334145	1.03947829434420	2.99257834403617
H	1.77226506639861	1.24713173099983	3.34867750177773
C	0.00094371650273	2.64140503573275	1.42258842204053
C	-0.00017527169437	3.98228793148813	1.94049929523711
H	-0.89754109027841	4.15088304943128	2.54446387648413
H	0.00140965390676	4.68487904009442	1.09910924496697
H	0.89456807279372	4.15143929956703	2.54816853261053
P	-1.57137757478781	-0.95895746881385	1.50128816560853
C	-0.66421636039331	-1.16725220944105	3.06719423725685
H	-1.24663172445086	-1.31433673090336	3.97750863343136
C	-2.16004820384052	-2.63807398829979	1.10016116347017
C	-1.83483298770713	-3.71564405807678	1.93334773928916
H	-1.22730974572442	-3.57136896532180	2.82003715042634
C	-2.31379514075570	-4.98943492018828	1.64393935993770
H	-2.07344879320502	-5.81497306542119	2.30683616639654
C	-3.11177175198073	-5.20165590959936	0.52422001112139
H	-3.49392862515669	-6.19521530950783	0.30972534814434
C	-3.42945755583731	-4.13380052632029	-0.31259483637609
H	-4.06128590574800	-4.28978057216465	-1.18216113439066
C	-2.95391488282351	-2.85817937154591	-0.03304953852765
H	-3.20826394868297	-2.04150045435480	-0.69873587235687
C	-3.00821941958045	0.00562015726189	2.07042120967616
C	-4.31385913731248	-0.27802637000726	1.65748480647716
H	-4.51764451959650	-1.10144125247342	0.98197731271784
C	-5.37309813265456	0.47904279111340	2.14883952008146
H	-6.38731857698047	0.24436671683704	1.84037699950046
C	-5.14202334837772	1.50951105677580	3.05537998840785
H	-5.97561258574514	2.08378920895344	3.44839194041520
C	-3.84400976163358	1.78133744070274	3.48514551287657
H	-3.66663405933008	2.55940098898902	4.22167444851068
C	-2.77846061302849	1.03959320435107	2.99221105981863
H	-1.77236388898747	1.24720911857794	3.34818074016813

Complex: $[Cu(CF_3)_4]^-$			
Cu	-0.00000382342215	-0.00000085332372	0.00000137403882
C	1.34820550968705	1.44791589443458	0.23771014258456
F	2.44348475399280	1.04776410790377	0.96327019078905
F	1.84065745965768	1.96392665714319	-0.92651458174724
F	0.86838371329035	2.52028716550464	0.94880498596636
C	-1.44791990978575	1.34820724507717	-0.23773309623319
F	-1.96393893547068	1.84067343505963	0.92648293954137
F	-1.04776477299796	2.44347836008108	-0.96330330488536
F	-2.52028567452018	0.86837692576908	-0.94883015215746
C	1.44791583644814	-1.34820558329331	-0.23771869416683
F	1.96388700010221	-1.84071199130184	0.92650058153168
F	1.04778859361763	-2.44345111454081	-0.96334282067800
F	2.52031100927041	-0.86835162366808	-0.94875581975926
C	-1.34820680547232	-1.44791880195908	0.23774235765669
F	-2.44346280754587	-1.04777234063544	0.96333906095629
F	-1.84069440764593	-1.96392256676007	-0.92647093182718
F	-0.86836381470543	-2.52029458429079	0.94881690078968

Energies (au)

E(PBE0/def2-TZVP//r^{2}SCAN-3c)
= −2990.181087539672

Thermal correction*
= 0.00919320

*** 1 atm, 298K, 1M, r^{2}SCAN-3c**

Complex: $RhCl(CO)(SO_2)(PPh_3)_2$			
Rh	-0.33557282123809	-0.19878970782955	-0.82349322763451
Cl	-2.67469650274216	0.19431049066461	-0.87670962546073
P	-0.48942318535379	0.05759666118955	1.53273538871203
P	-0.30393480125925	-0.11092843897937	-3.20003833312889
C	1.49390218635062	-0.02083498937793	-0.78227159306939
O	2.63050515844638	0.17370056026660	-0.77966538092360
S	0.05070003987590	-2.72249281756847	-0.88108333087103
O	0.46124158108056	-3.03941979224751	0.52015965853440
O	-1.33112050826254	-3.18715681592305	-1.23235193029485
C	0.84056275096666	-1.31882041782585	-3.97193956969543
C	2.22146244959840	-1.11133577169977	-3.86961456669345
C	0.36713746795923	-2.49277603000095	-4.56167195170218
C	3.10985690650091	-2.06079045260575	-4.35739721629272
C	1.26295124805283	-3.44249980162280	-5.04379297248454
C	2.63315421047989	-3.23115512246462	-4.94272769621902
H	2.60728788330116	-0.20210113134124	-3.41847618717707
H	-0.70007699893768	-2.66996321333814	-4.64784509212087
H	4.17861590412087	-1.88617333362647	-4.27540060904033
H	0.88239273984791	-4.35219884318234	-5.49865943928627
H	3.32956244944177	-3.97536069677474	-5.31788558066660
C	0.32534687154748	1.51886842047419	-3.74359554964168
C	0.14978553751450	2.61712520977322	-2.89814253503062
C	0.93923446724815	1.69622336034350	-4.98797044189132
C	0.58429519349692	3.87854945807911	-3.29111828822755
C	1.37475337315733	2.95848654351513	-5.37499847145607

Energies (au)

E(PBE0/def2-TZVP//r^{2}SCAN-3c)
= −3303.649641612707

Thermal correction*
= 0.49663054

*** 1 atm, 298K, 1M, r^{2}SCAN-3c**

C	1.19876423233555	4.04981603108150	-4.52762944490238	
H	-0.32506039271608	2.47663746781244	-1.92943463859653	
H	1.07954179727572	0.84575114807322	-5.64947568756220	
H	0.44796683161414	4.72585247023104	-2.62563733389768	
H	1.85445481090268	3.08962963977485	-6.34056626395905	
H	1.54425321187117	5.03380526108940	-4.83149493115749	
C	-1.88869688300199	-0.33951143935855	-4.08138133667564	
C	-2.71123092758351	-1.41116649079630	-3.71479081598276	
C	-2.28134964824546	0.51877647200611	-5.10992073593175	
C	-3.89835186814083	-1.63428081992302	-4.40109475664075	
C	-3.47832298758134	0.29276253974041	-5.78332138080843	
C	-4.28427466140407	-0.78544228648022	-5.43489927166482	
H	-2.43046341252153	-2.05822656351221	-2.88858108997227	
H	-1.66475506577829	1.36859891104406	-5.38484399719369	
H	-4.53218814365335	-2.46701354883933	-4.11118979630912	
H	-3.78011957905195	0.96690029740708	-6.57962487232376	
H	-5.21900548359891	-0.95770979071199	-5.96103689657135	
C	0.92503962680800	-0.58439399063053	2.50201732237108	
C	0.79603207175708	-1.73536728513221	3.28023532814294	
C	2.16902528479607	0.05015180390247	2.41270307084616	
C	1.89677310345652	-2.24001298943223	3.96449817869328	
C	3.26398170052686	-0.45599813081637	3.10053745367955	
C	3.13003446751197	-1.60421547719990	3.87739596053787	
H	-0.15970946386405	-2.24511250952563	3.34255669892890	
H	2.28329510261742	0.95050652212228	1.81532021593141	
H	1.78710987071697	-3.13972104982359	4.56254232299651	
H	4.22399756957120	0.04632404772063	3.02629236180539	
H	3.98789317735481	-2.00256843792591	4.41158624449787	
C	-1.95699644080163	-0.68204585791504	2.34098142747591	
C	-2.52343097074953	-1.84777889408616	1.82118189023671	
C	-2.47535919957290	-0.11679473037369	3.50871671979642	
C	-3.59181740609021	-2.44745145694570	2.47821527972608	
C	-3.54840988332673	-0.71985250519753	4.15596434983632	
C	-4.10586156574196	-1.88737673834270	3.64357327798649	
H	-2.14707001549475	-2.28654610660154	0.90368031903430	
H	-2.05192951875782	0.79887055415970	3.91072086880634	
H	-4.02797086674943	-3.35229886149377	2.06577532700590	
H	-3.95064097550072	-0.27154089097444	5.05981107363590	
H	-4.94617318562574	-2.35544737131583	4.14865408681547	
C	-0.54887603139256	1.84057789342947	1.98417461506963	
C	0.03032295117726	2.31859337532461	3.16504291637614	
C	-1.22457271691980	2.73256379561649	1.14464103142299	
C	-0.05758868019521	3.66781949378966	3.49280718908574	
C	-1.31082238262106	4.07944884570472	1.47979124696115	
C	-0.72462718707452	4.55162796302657	2.65027312307105	
H	0.55432193175919	1.63813196181036	3.82949066096861	
H	-1.70461136893405	2.36314823788131	0.24303249297199	
H	0.39723788951647	4.02647390392835	4.41171052886331	
H	-1.84321825270038	4.76070416526961	0.82216281371633	

References

[1] S. Grimme, A. Hansen, S. Ehlert, J.-M. Mewes, r^{2}SCAN-3c: A “Swiss army knife” composite electronic-structure method, *J. Chem. Phys.* **2021**, *154*, 064103. DOI: 10.1063/5.0040021

[2] F. Neese, Software update: the ORCA program system, version 4.0, *WIREs: Comput. Mol. Sci.* **2018**, *8*, e1327. DOI: 10.1002/wcms.1327

[3] F. Neese, The ORCA program system, *WIREs: Comput. Mol. Sci.* **2012**, *2*, 73. DOI: 10.1002/wcms.81

[4] C. Adamo, M. Cossi, V. Barone, An accurate density functional method for the study of magnetic properties: the PBE0 model, *J. Mol. Struct.: THEOCHEM* **1999**, *493*, 145. DOI: 10.1016/S0166-1280(99)00235-3

[5] F. Weigend, R. Ahlrichs, Balanced basis sets of split valence, triple zeta valence and quadruple zeta valence quality for H to Rn: Design and assessment of accuracy, *Phys. Chem. Chem. Phys.* **2005**, *7*, 3297. DOI: 10.1039/B508541A

[6] G. Knizia, Intrinsic Atomic Orbitals: An Unbiased Bridge between Quantum Theory and Chemical Concepts, *J. Chem. Theory Comput.* **2013**, *9*, 4834. DOI: 10.1021/ct400687b

[7] E. Ramos-Cordoba, V. Postils, P. Salvador, Oxidation States from Wave Function Analysis, *J. Chem. Theory Comput.* **2015**, *11*, 1501. DOI: 10.1021/ct501088v
[8] F. Neese, Importance of Direct Spin−Spin Coupling and Spin-Flip Excitations for the Zero-Field Splittings of Transition Metal Complexes: A Case Study, *J. Am. Chem. Soc.* **2006**, *128*, 10213. DOI: 10.1021/ja061798a
[9] J. G. Brandenburg, C. Bannwarth, A. Hansen, S. Grimme, B97-3c: A revised low-cost variant of the B97-D density functional method, *J. Chem. Phys.* **2018**, *148*, 064104. DOI: 10.1063/1.5012601
[10] I. F. Leach, D. Sorbelli, L. Belpassi, P. Belanzoni, R. W. A. Havenith, J. E. M. N. Klein, How reduced are nucleophilic gold complexes?, *Dalton Trans.* **2023**, *52*, 11. DOI: 10.1039/d2dt01694j
[11] Y. Zhao, D. G. Truhlar, The M06 suite of density functionals for main group thermochemistry, thermochemical kinetics, noncovalent interactions, excited states, and transition elements: two new functionals and systematic testing of four M06-class functionals and 12 other functionals, *Theor. Chem. Acc.* **2008**, *120*, 215. DOI: 10.1007/s00214-007-0310-x
[12] J. S. Steen, G. Knizia, J. E. M. N. Klein, σ-Noninnocence: Masked Phenyl-Cation Transfer at Formal Ni(IV), *Angew. Chem. Int. Ed.* **2019**, *58*, 13133. DOI: 10.1002/anie.201906658
[13] I. F. Leach, R. W. A. Havenith, J. E. M. N. Klein, Revisiting Formal Copper(III) Complexes: Bridging Perspectives with *Quasi-d^{10}* Configurations, *Eur. J. Inorg. Chem.* **2022**, *2022*, e202200247. DOI: 10.1002/ejic.202200247
[14] C. Adamo, V. Barone, Exchange functionals with improved long-range behavior and adiabatic connection methods without adjustable parameters: The mPW and mPW1PW models, *J. Chem. Phys.* **1998**, *108*, 664. DOI: 10.1063/1.475428
[15] K. A. Peterson, D. Figgen, M. Dolg, H. Stoll, Energy-consistent relativistic pseudopotentials and correlation consistent basis sets for the 4d elements Y–Pd, *J. Chem. Phys.* **2007**, *126*, 124101. DOI: 10.1063/1.2647019
[16] G. A. Petersson, M. A. Al-Laham, A complete basis set model chemistry. II. Open-shell systems and the total energies of the first-row atoms, *J. Chem. Phys.* **1991**, *94*, 6081. DOI: 10.1063/1.460447
[17] G. A. Petersson, A. Bennett, T. G. Tensfeldt, M. A. Al-Laham, W. A. Shirley, J. Mantzaris, A complete basis set model chemistry. I. The total energies of closed-shell atoms and hydrides of the first-row elements, *J. Chem. Phys.* **1988**, *89*, 2193. DOI: 10.1063/1.455064
[18] E. D. Litle, F. P. Gabbaï, Metal→Carbon Dative Bonding, *Angew. Chem. Int. Ed.* **2022**, *61*. DOI: 10.1002/anie.202201841
[19] A. D. Becke, Density-functional thermochemistry. I. The effect of the exchange-only gradient correction, *J. Chem. Phys.* **1992**, *96*, 2155. DOI: 10.1063/1.462066
[20] A. W. Ehlers, M. Böhme, S. Dapprich, A. Gobbi, A. Höllwarth, V. Jonas, K. F. Köhler, R. Stegmann, A. Veldkamp, G. Frenking, A set of f-polarization functions for pseudo-potential basis sets of the transition metals Sc–Cu, Y–Ag and La–Au, *Chem. Phys. Lett.* **1993**, *208*, 111. DOI: 10.1016/0009-2614(93)80086-5
[21] P. C. Hariharan, J. A. Pople, The influence of polarization functions on molecular orbital hydrogenation energies, *Theor. Chim. Acta* **1973**, *28*, 213. DOI: 10.1007/BF00533485

[22] M. Sircoglou, S. Bontemps, M. Mercy, N. Saffon, M. Takahashi, G. Bouhadir, L. Maron, D. Bourissou, Transition-Metal Complexes Featuring Z-Type Ligands: Agreement or Discrepancy between Geometry and d^n Configuration?*, Angew. Chem. Int. Ed.* **2007**, *46*, 8583. DOI: 10.1002/anie.200703518

[23] D. A. Kurtz, J. Zhang, A. Sookezian, J. Kallick, M. G. Hill, B. M. Hunter, A Cobalt Phosphine Complex in Five Oxidation States*, Inorg. Chem.* **2021**, *60*, 17445. DOI: 10.1021/acs.inorgchem.1c03020

[24] I. F. Leach, *d8 Cu(III) vs. d10 Cu(I): a debate as old as I…*, accessed 15/11/2023, isaacfleach.wordpress.com/2022/08/27/d8-cuiii-vs-d10-cui-a-debate-as-old-as-i/

[25] R. Hoffmann, S. Alvarez, C. Mealli, A. Falceto, T. J. Cahill, T. Zeng, G. Manca, From Widely Accepted Concepts in Coordination Chemistry to Inverted Ligand Fields*, Chem. Rev.* **2016**, *116*, 8173. DOI: 10.1021/acs.chemrev.6b00251

[26] I. M. DiMucci, J. T. Lukens, S. Chatterjee, K. M. Carsch, C. J. Titus, S. J. Lee, D. Nordlund, T. A. Betley, S. N. MacMillan, K. M. Lancaster, The Myth of d^8 Copper(III)*, J. Am. Chem. Soc.* **2019**, *141*, 18508. DOI: 10.1021/jacs.9b09016

[27] R. C. Walroth, J. T. Lukens, S. N. MacMillan, K. D. Finkelstein, K. M. Lancaster, Spectroscopic Evidence for a $3d^{10}$ Ground State Electronic Configuration and Ligand Field Inversion in $[Cu(CF_3)_4]^{1-}$*, J. Am. Chem. Soc.* **2016**, *138*, 1922. DOI: 10.1021/jacs.5b10819

[28] I. M. Dimucci, C. J. Titus, D. Nordlund, J. R. Bour, E. Chong, D. P. Grigas, C.-H. Hu, M. D. Kosobokov, C. D. Martin, L. M. Mirica, N. Nebra, D. A. Vicic, L. L. Yorks, S. Yruegas, S. N. Macmillan, J. Shearer, K. M. Lancaster, Scrutinizing formally Ni^{IV} centers through the lenses of core spectroscopy, molecular orbital theory, and valence bond theory*, Chem. Sci.* **2023**, *14*, 6915. DOI: 10.1039/d3sc02001k

[29] A. Ienco, F. Ruffo, G. Manca, The Role of Inverted Ligand Field in the Electronic Structure and Reactivity of Octahedral Formal Platinum (IV) Complexes***, Chem. Eur. J.* **2023**, *29*, e202301669. DOI: 10.1002/chem.202301669
